# Regional differences in cancer treatment among American Indian and Alaska native peoples: a surveillance, epidemiology, and end results study

**DOI:** 10.1007/s10552-026-02271-9

**Published:** 2026-11-03

**Authors:** Faith Elenwa, Jennifer Erdrich, Jamie Sorensen, Vanessa E. Slater, Carol Goldtooth, Priscilla R. Sanderson, Tamee Livermont, Keely Ulmer, Bradley D. McDowell, Sarah H. Nash,

**Affiliations:** 1 Department of Epidemiology, College of Public Health, University of Iowa, Iowa City, IA USA; 2 Department of Surgery, University of Arizona, Tucson, AZ USA; 3 Department of Epidemiology, Gillings School of Global Public Health, University of North Carolina, Chapel Hill, NC USA; 4 Holden Comprehensive Cancer Center, University of Iowa, Iowa City, IA USA; 5 Monument Health, Rapid City, SD USA; 6 New Mexico Tumor Registry, University of New Mexico Comprehensive Cancer Center, Bldg 229, 1, Albuquerque, NM MSC11 602087131-0001 USA; 7 Partnership for Native American Cancer Prevention, Northern Arizona University, Arizona, USA; 8 Health Sciences Department, College of Health and Human Services, Northern Arizona University, Flagstaff, AZ USA; 9 University of California San Francisco East Bay General Surgery Residency, San Francisco, CA USA

**Keywords:** Health Equity, SEER, Native American, Indian Health Service

## Abstract

**Purpose:**

To determine whether regional variation in diagnostic procedures and treatment for cancer exists among American Indian and Alaska Native (AIAN) patients.

**Methods:**

We used data from the Surveillance Epidemiology and End Results (SEER) 17 database to examine differences in diagnostic, treatment, and care quality-related variables for female breast, lung, and colorectal cancers diagnosed among AIAN patients. We used unadjusted and multivariable-adjusted logistic regression models to examine differences across Indian Health Service  regions adjusted for age at diagnosis, diagnosis year, residence in a purchased/referred care delivery area, and stage at diagnosis, using the Pacific Coast region as the reference.

**Results:**

The study sample included female breast (n=7,221), lung (n=5,403), and colorectal cancer (n=5,626) patients. Among AIAN female breast cancer patients, those in Alaska, the East, and the Southwest had higher odds of timely treatment; those in Alaska had higher odds of receipt of chemotherapy and hormone receptor testing; while those in the Southwest had lower odds of hormone receptor testing. For lung cancer patients, those in Alaska, the Southwest, and the East had higher odds of timely treatment; those in Alaska had higher odds of receiving radiation and lower odds of receiving surgery. Finally, for colorectal cancer patients, those in Alaska, the Southwest, and the East had higher odds of receiving timely treatment.

**Conclusions:**

Differences described herein may reflect differences in clinical characteristics, practice patterns, or access to care. Further research on how treatment differences lead to regional variations in cancer outcomes for AIAN patients are warranted.

**Supplementary Information:**

The online version contains supplementary material available at https://doi.org/10.1007/s10552-026-02271-9.

## Introduction

American Indian and Alaska Native (AIAN) peoples have a unique burden of cancer, including higher incidence and mortality of several cancers (e.g., stomach, liver, lung), and lower cancer survival [1–4]. Receipt of timely and guideline concordant cancer treatment is both a hallmark of quality cancer care and a predictor of cancer survival [5]. AIAN patients experience greater cancer treatment delays and are less likely to receive guideline concordant care than their non-Hispanic white (NHW) counterparts [6, 7]. This lack of guideline concordant care receipt may contribute to observed survival disparities, since health services and clinical care are estimated to contribute approximately 20% to modifiable health outcomes [8]. Critically, while cancer survival is lower among AIAN patients not receiving guideline-concordant cancer care, when AIAN patients do receive guideline-concordant treatment, cancer-specific survival does not differ from that observed among NHW [6]. Therefore, understanding access to and receipt of cancer care among AIAN patients may shed light on ways to address cancer disparities affecting this population.

Given the heterogeneity of AIAN cultures, cancer burden, and the regional provision of healthcare through the Indian Health Service (IHS) and/or Tribally-managed care, it will be impractical to design and implement national-level interventions for AIAN peoples. Rather, there is a need to understand cancer care delivery at the regional level to determine how and where to partner with AIAN communities that could particularly benefit from health system-level interventions. This need is supported by regional differences in cancer burden: while all-sites cancer incidence is highest among AIAN people in the Northern Plains (666.7/100,000 population) and lowest in the Southwest (323.2), patterns are different for the leading cancers [1]. For example, female breast cancer incidence is highest in the Southern Plains (166.8) and lowest in the Southwest (69.9); Alaska Native people have among the highest incidence of (91.3) and mortality from (37.4) colorectal cancer globally, while rates are lowest in the East (34.2); and, lung cancer incidence is highest in the Northern Plains (109.3), and remarkably low in the Southwest (16.8), due to low prevalence of smoking [1, 9]. Variations in mortality and survival are less well studied; however, one study has shown differences in survival from the leading cancers (i.e., female breast, lung, and colorectal cancers) by region [10]. Further, there is good reason to expect regional differences in access to cancer care among AIAN patients, since IHS funds are managed at the regional level, and the extent to which Tribes contract with IHS or directly manage healthcare for their people also varies by region [1, 11, 12].

In this study, we investigated regional differences in cancer diagnostic and treatment procedures recorded for leading cancers (i.e., female breast, lung, and colorectal cancers) among AIAN patients using data from the Surveillance, Epidemiology, and End Results (SEER) Program. Further, as a secondary analysis to provide context for regional differences among AIAN patients, we compared treatments received and timely care among AIAN patients combined to a matched sample of non-Hispanic whites.

## Methods

### Ethics and data availability

This University of Iowa Institutional Review Board determined that this study did not meet the definition of human subjects research. Data are available through application to the NCI’s SEER Program.

### Data source and study population

This retrospective cohort study used data from 17 SEER Program Registries [13]. All AIAN patients aged 20+ years with a first primary breast (female only, [International Classification of Diseases – Oncology codes C500-509]), colorectal (C180, C182-189, C260, C199, C209) or lung malignancy (C340-349) diagnosed between 2000 and 2021 were eligible for inclusion in this study. We created three analytic cohorts, one for each cancer site included: female breast, lung, and colorectal cancers. While our primary focus was on regional differences among AIAN patients within these three cohorts, we also compared all AIAN patients to NHW, to provide a benchmark for contextualizing within-AIAN differences. Thus, we created a second series of cohorts, defined identically by cancer site, but including both AIAN and NHW patients; a description of the matching process is described below.

### Exposure and outcomes

For full details on variable and strata definitions, see Supplementary Table 1. The primary exposure of interest in this study was IHS region, categorized as East (Kentucky, Georgia, Connecticut, New Jersey, Louisiana), Northern Plains (Iowa), Southwest (New Mexico, Utah), Pacific Coast (California, Hawaii, Seattle), and Alaska (Alaska Native Tumor Registry), defined using the SEER Registry variable and classified according to IHS definitions [3]. There were no SEER registries that represented the Southern Plains states in the database; therefore, this region could not be included in our analysis. The exposure in our secondary analysis was race: we included only AIAN and NHW patients in this analysis.

Outcome measures varied by cancer site. For all cancer sites, we examined time to treatment initiation stratified as <= 60 and > 60 days, among those who received treatment. This time-point was chosen based on studies that indicate treatment initiation greater than 60 days post-diagnosis may increase risk of death, including for those cancer sites explored herein [14, 15]. Those who did not receive treatment were excluded from these analyses, as we could not definitively determine whether lack of treatment was due to patient choice or health system-related delays (the latter only indicating a lack of “high-quality care”). For each cancer site, we defined microscopic confirmation as Yes/No, and late-stage diagnosis included those diagnosed at regional or distant stage. Receipt of surgery, radiation therapy and chemotherapy were defined as Yes/No. Completeness of radiation and chemotherapy variables in SEER Program registries is known to be low: one study comparing SEER to Medicare data observed 68% and 80% sensitivity for receipt of chemotherapy and radiation (overall), respectively [16]. For this reason, it is not recommended for use in estimating prevalence of chemotherapy or radiation services received. We include these variables in this study with the assumption that missingness of these data are non-differential by IHS region or race; therefore, are suitable to indicate relative receipt (e.g., ranking regions with the highest % radiation received is likely to represent “truth,” even if the proportions themselves are lower than “truth”).

For female breast cancers only, we examined receipt of breast conserving surgery documented among those who had surgery. We defined occurrence of definitive HER2 and ER/PR testing as Yes (for Negative or Positive results), and No (for Borderline/Unknown results). Patients had to have valid results for both ER and PR tests to be considered tested, as a borderline result was considered non-informative for clinical practice (i.e., treatment decision-making). For colorectal cancer cases, we examined surgery received, among all patients and stratified by cancer site. We additionally examined KRAS documentation (AJCC stage IV cases only; 2018 cases onwards), categorized as Yes or No. Microsatellite Instability (MSI) testing documented was categorized using the MSI variable (2018 cases and onwards) as Yes or No. Whether 12+ Regional Nodes Examined was categorized using the Regional Lymph Nodes Examined variable as Yes or No.

### Covariates

For full details on variable and strata definitions, see Supplementary Table 1. Covariates included sex (stratified as male, female; colorectal and lung cancers only), age at diagnosis (stratified as stratified as 20–39 years, 40–64 years, and 65+ years), year of diagnosis (2000–2005, 2006–2010, 2011–2015, and 2016–2021), ethnicity (Hispanic, non-Hispanic), PRCDA residence (yes/no), rurality (rural/urban), marital status (partnered, not partnered, unknown), median household income (< $40,000, $40,000-$99,999, >$100,000), cancer stage (local, regional, distant, unknown), and treatment at a Commission on Cancer (CoC) accredited facility (yes/no). Each SEER registry routinely links with the IHS to improve quality of AIAN race reporting [17]; however, AIAN misclassification is higher in non-Purchased/Referred Care Delivery Areas (PRCDA) because the IHS linkage is less effective in these counties. Often, these cases are excluded from analyses [2–4, 18–20]. However, we decided to include all AIAN patients, regardless of PRCDA residence, in this analysis so as not to cause data erasure for those residing in a non-PRCDA county [21], and to be as inclusive as possible. We used the Rural Urban Continuum Codes (RUCC) to categorize rurality; since the Alaska Native Tumor Registry does not share geocoding data with the SEER Program, all cases in Alaska were coded Rural by the SEER Program. Treatment at a CoC facility was determined based on the assumption that an analytic abstract indicates treatment at the CoC accredited facility (i.e., as opposed to diagnosis only).

### Comparisons between AIAN and NHW peoples

Due to the large differences in case counts between AIAN peoples and NHW, we conducted a 1:10 matching of AI/AN patients to NHW patients based on exact age and 5-year diagnosis year group without replacement. Within each exact age-year of diagnosis stratum, we selected up to ten NHW patients per AI/AN patient. The analytic cohort consisted of only matched patients. Statistical analyses were conducted as described below; the key difference between these and the primary analyses was that the exposure variable was race, rather than region.

### Statistical analysis

We assessed continuous variables for normality using the Shapiro–Wilk test. We present normally distributed variables as mean ± standard deviation (SD), and non-normally distributed variables as median with interquartile range (IQR, Q1–Q3). Categorical variables were summarized as counts and proportions (%). We conducted descriptive analyses, with differences between groups assessed using Chi-squared tests for categorical variables or ANOVA for continuous variables. We conducted unadjusted and multivariable-adjusted logistic regression models to assess associations between IHS region and treatment received. Adjusted models were adjusted for diagnosis year, sex (colorectal and lung cancers only), age group, ethnicity, marital status at diagnosis, stage (except for the model in which the outcome was diagnose at a late stage), and residence in a PRCDA county at time of diagnosis. Final models were selected using a Directed Acyclic Graph approach and based on data availability. Models used different sample populations based on the years of data available for each outcome; analytic sample size is noted in table footnotes where they differ from the main sample. For breast cancer only, we conducted a sensitivity analysis including only breast cancer cases diagnosed in 2010 onwards, where we also adjusted for breast cancer receptor subtype (except for the model where the outcome was ER/PR testing occurred). Odds ratios (ORs) and 95.0% confidence intervals (95.0% CI) are reported. A p-value < 0.05 was considered statistically significant. All statistical analyses were performed using SAS 9.4 (SAS Institute, Cary, North Carolina, USA) and R v4.2.1.

## Results

### Demographic and clinical characteristics: female breast cancers

Demographic and clinical characteristics of female breast cancer patients (*n* = 7,221) are given in Table 1. Characteristics of the sample restricted just to those with subtype information is given in Supplementary Table 2. Mean age at diagnosis varied from 57.5 years (±11.8) in the Northern Plains and Alaska regions to 59.2 years (±13.0) in the Pacific Coast region. Stage distribution also varied by region, with diagnosis at distant stage varying from 5.8% in Alaska to 8.1% in the Northern Plains. There were also variations in breast subtype by region; for example, the proportion of women with triple negative breast cancers (i.e., HR-/HER2-) ranged from 9.7% in Alaska to 14.9% in the Northern Plains.

**Table 1:** Characteristics of AIAN people with breast cancer (females only), stratified by IHS region

Category	Alaska (*n* = 1340)	East (*n* = 532)	Northern Plains (*n* = 100)	Pacific Coast (*n* = 3820)	Southwest (*n* = 1429)
Diagnosis age (mean (SD))					
	57.5 (12.7)	58.3 (13.8)	57.5 (11.8)	59.2 (13)	58 (13.1)
Age group (%)					
20–39	100 (7.5%)	41 (7.7%)	<5	244 (6.4%)	109 (7.6%)
40–64	845 (63.1%)	314 (59%)	69 (69%)	2216 (58%)	878 (61.4%)
65+	395 (29.5%)	177 (33.3%)	27 (27%)	1360 (35.6%)	442 (30.9%)
Income (%)					
< $40,000	0 (0%)	6 (1.1%)	0 (0%)	0 (0%)	154 (10.8%)
$40,000–100,000	1340 (100%)	449 (84.4%)	100 (100%)	3345 (87.6%)	1264 (88.5%)
>= $100,000	0 (0%)	77 (14.5%)	0 (0%)	475 (12.4%)	7 (0.5%)
Missing	0 (0%)	0 (0%)	0 (0%)	0 (0%)	<5
Ethnicity (%)					
Non-Hispanic	1339 (99.9%)	489 (91.9%)	98 (98%)	3454 (90.4%)	1335 (93.4%)
Hispanic	<5	43 (8.1%)	<5	366 (9.6%)	94 (6.6%)
Marital status at diagnosis (%)					
Not Partnered	431 (32.2%)	224 (42.1%)	53 (53%)	1776 (46.5%)	636 (44.5%)
Partnered	595 (44.4%)	283 (53.2%)	41 (41%)	1820 (47.6%)	558 (39%)
Unknown	314 (23.4%)	25 (4.7%)	6 (6%)	224 (5.9%)	235 (16.4%)
RUCC (%)					
Rural	1340 (100%)	69 (13%)	44 (44%)	549 (14.4%)	591 (41.4%)
Urban	0 (0%)	463 (87%)	56 (56%)	3271 (85.6%)	838 (58.6%)
Diagnosis year (%)					
2000–2005	339 (25.3%)	75 (14.1%)	19 (19%)	727 (19%)	277 (19.4%)
2006–2010	263 (19.6%)	107 (20.1%)	16 (16%)	785 (20.5%)	292 (20.4%)
2011–2015	339 (25.3%)	117 (22%)	27 (27%)	952 (24.9%)	367 (25.7%)
2016–2021	399 (29.8%)	233 (43.8%)	38 (38%)	1356 (35.5%)	493 (34.5%)
PRCDA (%)					
Yes	1340 (100%)	98 (18.4%)	41 (41%)	2771 (72.5%)	1389 (97.2%)
No	0 (0%)	434 (81.6%)	59 (59%)	1049 (27.5%)	40 (2.8%)
CoC accredited flag (%)^a^					
Yes	20 (7.1%)	140 (84.3%)	18 (75%)	725 (76.4%)	219 (65%)
No	8 (2.8%)	9 (5.4%)	0 (0%)	47 (5%)	18 (5.3%)
Grade (%)^b^					
Well, differentiated	353 (26.3%)	90 (16.9%)	21 (21%)	770 (20.2%)	241 (16.9%)
Moderately differentiated	485 (36.2%)	203 (38.2%)	35 (35%)	1531 (40.1%)	491 (34.4%)
Poorly differentiated	377 (28.1%)	190 (35.7%)	36 (36%)	1219 (31.9%)	478 (33.4%)
Undifferentiated	<5	<5	<5	31 (0.8%)	6 (0.4%)
Unknown	121 (9%)	46 (8.6%)	7 (7%)	269 (7%)	213 (14.9%)
Stage (%)^c^					
Localized	706 (62.9%)	286 (58.8%)	57 (66.3%)	2005 (60%)	721 (57%)
Regional	343 (30.5%)	161 (33.1%)	21 (24.4%)	1067 (31.9%)	403 (31.9%)
Distant	65 (5.8%)	30 (6.2%)	7 (8.1%)	203 (6.1%)	100 (7.9%)
Unknown/Unstaged	9 (0.8%)	9 (1.9%)	<5	65 (1.9%)	41 (3.2%)
Breast cancer subtype (%)^d^					
HR+/HER2+	99 (12.4%)	37 (9.8%)	8 (11.9%)	253 (10.2%)	115 (12.4%)
HR+/HER2-	548 (68.8%)	263 (69.4%)	45 (67.2%)	1709 (68.9%)	563 (60.9%)
HR-/HER2+	45 (5.7%)	19 (5%)	<5	114 (4.6%)	42 (4.5%)
HR-/HER2-	77 (9.7%)	38 (10%)	10 (14.9%)	251 (10.1%)	93 (10.1%)
No/Unknown	27 (3.4%)	22 (5.8%)	<5	154 (6.2%)	111 (12%)

Data from the surveillance, epidemiology, and end results program, 2000–2021, *n* = 7,221

^a^CoC Accredited Flag from 2018 onwards. A total of 1,204 individuals were included in this analysis

^b^From 2000 to 2017, variable Grade Recode was used to define grade; from 2018 to present, pathological grade was used

^c^SEER derived summary stage from 2004 onwards. A total of 6,300 individuals were included in this analysis

^d^Subtype from 2010 onwards. A total of 4,647 individuals were included in this analysis

### Differences in components of care received by region, female breast cancers

**Fig. 1:**
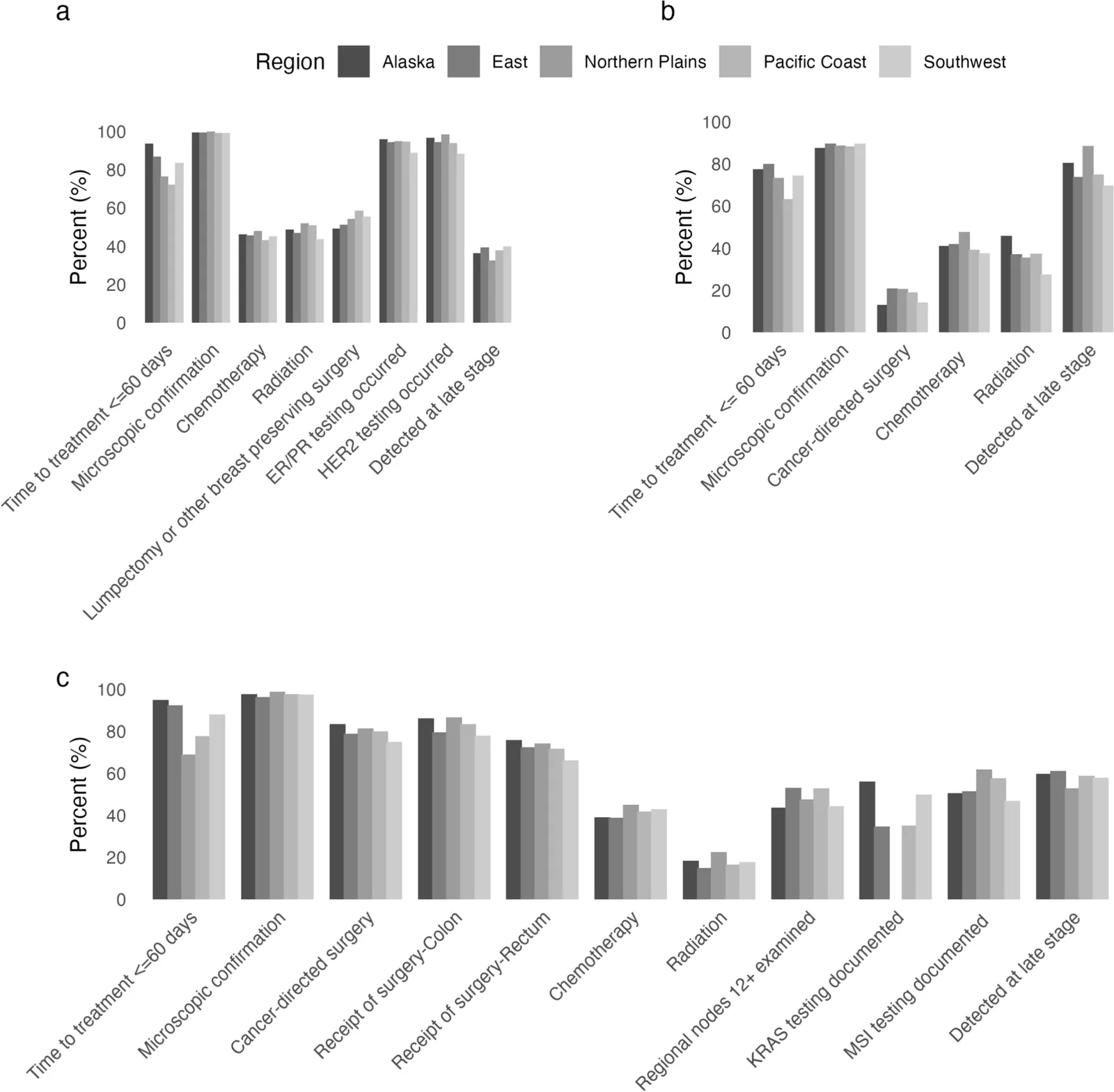
Percentage of individuals receiving components of care among AIAN people with **A** breast (female only), **B** lung, and **C** colorectal cancer, by region, 2000–2021. Data from the Surveillance, Epidemiology, and End Results program

Frequencies and proportions of female patients with breast cancer receiving each component of care by region are shown in Fig. 1a and given in Supplementary Table 3 (see Supplementary Table 4 for information restricted to the subset of patients with subtype information). Among the full cohort, time to treatment ranged from a median of 22 days (13, 34) in Alaska to 32 days (17, 50) on the Pacific Coast. The proportion of patients receiving treatment within 60 days ranged from 72.3% in the Pacific Coast region, to 93.6% in Alaska. Testing for hormone receptor (i.e., ER/PR) and HER2 status was high but not universal, ranging from 88.9% in the Southwest to 95.9% in Alaska for ER/PR testing, and 88.3% in the Southwest to 98.5% in the Northern Plains for HER2 testing. Types of surgery varied by region. The proportion of women receiving lumpectomy or other breast preserving surgery, for example, varied from 49.3% in Alaska, to 56.6% in the Southwest. Receipt of radiation and chemotherapy differed little by region (Tables 2, 3).

**Table 2:** Characteristics of AIAN people with lung cancer, stratified by IHS region

Category	Alaska (*n* = 1423)	East (*n* = 449)	Northern Plains (*n* = 107)	Pacific Coast (*n* = 2957)	Southwest (*n* = 467)
Diagnosis age (mean (SD))					
	68.0 (10.1)	66.6 (10.9)	68.0 (10.2)	67.9 (10.6)	69.6 (12.2)
Age group (%)					
20–39	5 (0.4%)	6 (1.3%)	<5	18 (0.6%)	11 (2.4%)
40–64	518 (36.4%)	180 (40.1%)	39 (36.4%)	1103 (37.3%)	131 (28.1%)
65+	900 (63.2%)	263 (58.6%)	67 (62.6%)	1836 (62.1%)	325 (69.6%)
Sex (%)					
Male	768 (54%)	242 (53.9%)	45 (42.1%)	1425 (48.2%)	262 (56.1%)
Female	655 (46%)	207 (46.1%)	62 (57.9%)	1532 (51.8%)	205 (43.9%)
Income (%)					
< $40,000	0 (0%)	9 (2%)	0 (0%)	0 (0%)	41 (8.8%)
$40,000–100,000	1423 (100%)	403 (89.8%)	107 (100%)	2701 (91.3%)	420 (89.9%)
>= $100,000	0 (0%)	37 (8.2%)	0 (0%)	256 (8.7%)	<5
Missing	0 (0%)	0 (0%)	0 (0%)	0 (0%)	<5
Ethnicity (%)					
Non-Hispanic	1421 (99.9%)	421 (93.8%)	104 (97.2%)	2814 (95.2%)	431 (92.3%)
Hispanic	<5	28 (6.2%)	<5	143 (4.8%)	36 (7.7%)
Marital status at diagnosis (%)					
Not partnered	534 (37.5%)	233 (51.9%)	64 (59.8%)	1652 (55.9%)	201 (43%)
Partnered	536 (37.7%)	193 (43%)	42 (39.3%)	1176 (39.8%)	203 (43.5%)
Unknown	353 (24.8%)	23 (5.1%)	<5	129 (4.4%)	63 (13.5%)
RUCC (%)					
Rural	1423 (100%)	85 (18.9%)	36 (33.6%)	574 (19.4%)	208 (44.5%)
Urban	0 (0%)	364 (81.1%)	71 (66.4%)	2383 (80.6%)	259 (55.5%)
Diagnosis year (%)					
2000–2005	345 (24.2%)	90 (20%)	19 (17.8%)	643 (21.7%)	96 (20.6%)
2006–2010	310 (21.8%)	87 (19.4%)	24 (22.4%)	630 (21.3%)	96 (20.6%)
2011–2015	314 (22.1%)	111 (24.7%)	32 (29.9%)	726 (24.6%)	108 (23.1%)
2016–2021	454 (31.9%)	161 (35.9%)	32 (29.9%)	958 (32.4%)	167 (35.8%)
PRCDA (%)					
Yes	1423 (100%)	83 (18.5%)	39 (36.4%)	2342 (79.2%)	436 (93.4%)
No	0 (0%)	366 (81.5%)	68 (63.6%)	615 (20.8%)	31 (6.6%)
CoC accredited flag (%)					
Yes	11 (3.6%)	79 (71.2%)	14 (63.6%)	477 (69.5%)	75 (67.6%)
No	9 (2.9%)	15 (13.5%)	0 (0%)	77 (11.2%)	7 (6.3%)
Grade (%)					
Well, differentiated	58 (4.1%)	11 (2.4%)	<5	134 (4.5%)	35 (7.5%)
Moderately differentiated	160 (11.2%)	77 (17.1%)	17 (15.9%)	380 (12.9%)	58 (12.4%)
Poorly differentiated	280 (19.7%)	83 (18.5%)	23 (21.5%)	642 (21.7%)	76 (16.3%)
Undifferentiated	40 (2.8%)	21 (4.7%)	8 (7.5%)	109 (3.7%)	8 (1.7%)
Unknown	885 (62.2%)	257 (57.2%)	58 (54.2%)	1692 (57.2%)	290 (62.1%)
Stage (%)					
Localized	197 (15.7%)	88 (21.4%)	9 (9.4%)	521 (19.8%)	77 (18.5%)
Regional	299 (23.9%)	95 (23.1%)	24 (25%)	577 (21.9%)	70 (16.8%)
Distant	711 (56.7%)	209 (50.7%)	61 (63.5%)	1399 (53.1%)	220 (52.9%)
Unknown/Unstaged	46 (3.7%)	20 (4.9%)	<5	137 (5.2%)	49 (11.8%)

Data from the Surveillance, Epidemiology, and End Results program, 2000–2021, n = 5,403

^a^CoC Accredited Flag from 2018 onwards. A total of 764 participants were included

^b^From 2000 to 2017, variable Grade Recode was used to define grade; from 2018 to present, pathological grade was used

^c^SEER derived summary stage from 2004 onwards. A total of 4,811 participants were included

**Table 3:** Characteristics of AIAN people with colorectal cancer, stratified by IHS region

Category	Alaska (*n* = 1472)	East (*n* = 355)	Northern Plains (*n* = 80)	Pacific Coast (*n* = 2379)	Southwest (*n* = 1340)
Diagnosis age (mean (SD))					
	63 (13.5)	63.3 (13)	64.8 (13.1)	63.3 (13.5)	61.2 (14.3)
Age group (%)					
20–39	66 (4.5%)	15 (4.2%)	<5	108 (4.5%)	104 (7.8%)
40–64	719 (48.8%)	166 (46.8%)	37 (46.3%)	1128 (47.4%)	680 (50.7%)
65+	687 (46.7%)	174 (49%)	40 (50%)	1143 (48%)	556 (41.5%)
Sex (%)					
Male	706 (48%)	195 (54.9%)	40 (50%)	1193 (50.1%)	704 (52.5%)
Female	766 (52%)	160 (45.1%)	40 (50%)	1186 (49.9%)	636 (47.5%)
Income (%)					
< $40,000	0 (0%)	6 (1.7%)	0 (0%)	0 (0%)	161 (12%)
$40,000–100,000	1472 (100%)	302 (85.1%)	80 (100%)	2118 (89%)	1169 (87.2%)
>= $100,000	0 (0%)	47 (13.2%)	0 (0%)	261 (11%)	6 (0.4%)
Missing	0 (0%)	0 (0%)	0 (0%)	0 (0%)	<5
Ethnicity (%)					
Non-Hispanic	1467 (99.7%)	336 (94.6%)	75 (93.8%)	2133 (89.7%)	1235 (92.2%)
Hispanic	5 (0.3%)	19 (5.4%)	5 (6.3%)	246 (10.3%)	105 (7.8%)
Marital status at diagnosis (%)					
Not partnered	426 (28.9%)	160 (45.1%)	47 (58.8%)	1212 (50.9%)	563 (42%)
Partnered	596 (40.5%)	167 (47%)	28 (35%)	1015 (42.7%)	570 (42.5%)
Unknown	450 (30.6%)	28 (7.9%)	5 (6.3%)	152 (6.4%)	207 (15.4%)
RUCC (%)					
Rural	1472 (100%)	63 (17.7%)	34 (42.5%)	404 (17%)	679 (50.7%)
Urban	0 (0%)	292 (82.3%)	46 (57.5%)	1975 (83%)	661 (49.3%)
Diagnosis year (%)					
2000–2005	345 (23.4%)	41 (11.5%)	17 (21.3%)	467 (19.6%)	221 (16.5%)
2006–2010	307 (20.9%)	75 (21.1%)	13 (16.3%)	493 (20.7%)	256 (19.1%)
2011–2015	353 (24%)	103 (29%)	17 (21.3%)	602 (25.3%)	372 (27.8%)
2016–2021	467 (31.7%)	136 (38.3%)	33 (41.3%)	817 (34.3%)	491 (36.6%)
PRCDA (%)					
Yes	1472 (100%)	70 (19.7%)	38 (47.5%)	1809 (76%)	1306 (97.5%)
No	0 (0%)	285 (80.3%)	42 (52.5%)	570 (24%)	34 (2.5%)
CoC accredited flag (%)					
Yes	14 (4.5%)	73 (73.7%)	<5	406 (68.2%)	233 (67.1%)
No	0 (0%)	7 (7.1%)	<5	33 (5.5%)	19 (5.5%)
Grade (%)					
Well, differentiated	199 (13.5%)	30 (8.5%)	9 (11.3%)	239 (10%)	184 (13.7%)
Moderately differentiated	865 (58.8%)	189 (53.2%)	42 (52.5%)	1390 (58.4%)	683 (51%)
Poorly differentiated	129 (8.8%)	56 (15.8%)	10 (12.5%)	314 (13.2%)	149 (11.1%)
Undifferentiated	16 (1.1%)	10 (2.8%)	<5	36 (1.5%)	12 (0.9%)
Unknown	263 (17.9%)	70 (19.7%)	15 (18.8%)	400 (16.8%)	312 (23.3%)
Stage (%)					
Localized	501 (38%)	114 (33.5%)	30 (41.7%)	789 (36%)	431 (34.5%)
Regional	471 (35.7%)	119 (35%)	25 (34.7%)	801 (36.6%)	437 (34.9%)
Distant	317 (24%)	89 (26.2%)	13 (18.1%)	489 (22.3%)	288 (23%)
Unknown/Unstaged	31 (2.3%)	18 (5.3%)	<5	112 (5.1%)	95 (7.6%)

Data from the Surveillance, Epidemiology, and End Results program, 2000–2021, n = 5,626

^a^CoC Accredited Flag from 2018 onwards. A total of 791 individuals were included

^b^From 2000 to 2017, variable Grade Recode was used to define grade; from 2018 to present, pathological grade was used

^c^SEER derived summary stage from 2004 onwards. A total of 5,174 individuals were included

**Fig. 2:**
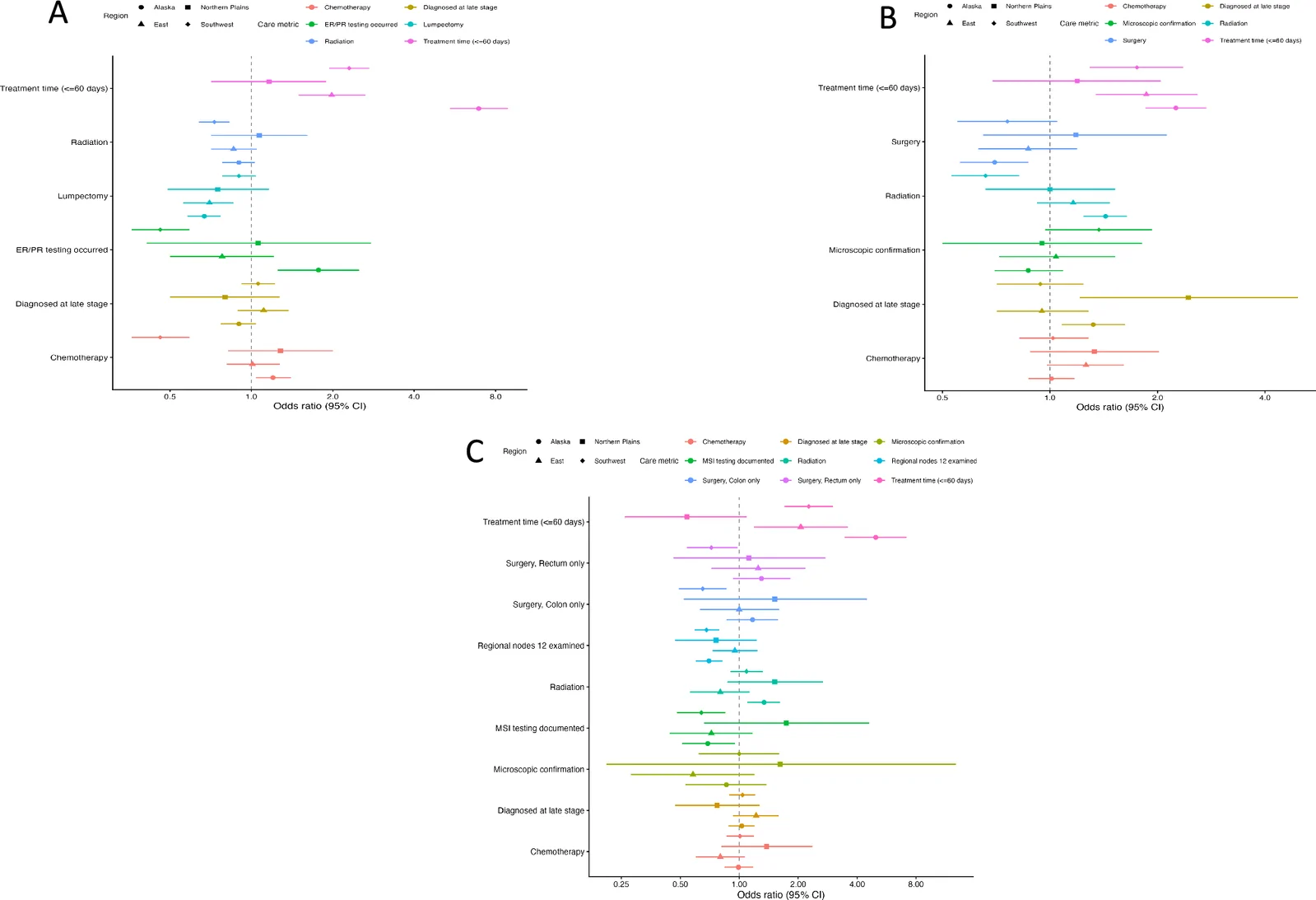
Forest plot shoring multivariable-adjusted associations of region with component of care among AIAN people with **A** breast (female only), **B** lung, and **C** colorectal cancer, by region, 2000–2021. Data from the Surveillance, Epidemiology, and End Results program

Unadjusted and adjusted associations of region with component of breast cancer care are given in Table 4; a forest plot showing the primary, multivariable-adjusted associations is given in Fig. 2a. Full results of the adjusted models are given in Supplementary Table 5, and a sensitivity analysis showing full models run only on individuals for which we were able to adjust for breast cancer subtype (i.e., 2010 onwards, n = 4,647) is given in Supplementary Table 6. After adjustment for age at diagnosis, diagnosis year, PRCDA, ethnicity, marital status, and stage, the primary differences observed were in time to treatment <=60 days. Women in Alaska (OR 6.94 [5.42, 8.89]), the East (OR 1.98 [1.49, 2.64]), and the Southwest (OR 2.30 [1.94, 2.73]) had higher odds of timely treatment than those in the Pacific Coast. We also observed that women in Alaska had higher odds, and women in the Southwest had lower odds, of both ER/PR and HER2 testing, compared to those in the Pacific Coast. There were no differences in odds of late-stage diagnosis by region. Results were similar when restricting to 2010 onwards and adjusting for breast subtype.

**Table 4:** Unadjusted and multivariable adjusted associations of region with components of treatment received for breast cancer among AIAN women, Surveillance, Epidemiology and Results Program, 2000–2021, n= 7,221

Region^a^	Treatment Time (< = 60 days)^b^	ER/PR testing occurred	Lumpectomy^c^	Chemotherapy	Radiation	Diagnosed at late stage^d^
Unadjusted						
Alaska	**5.60 (4.43, 7.09)**	1.32 (0.97, 1.79)	**0.69 (0.60, 0.78)**	1.12 (0.99, 1.27)	0.91 (0.80, 1.03)	0.91 (0.79, 1.05)
East	**2.52 (1.92, 3.29)**	0.94 (0.64, 1.40)	**0.74 (0.61, 0.90)**	1.10 (0.92, 1.32)	0.85 (0.71, 1.02)	1.05 (0.87, 1.28)
Northern Plains	1.25 (0.77, 2.03)	1.07 (0.43, 2.66)	0.84 (0.55, 1.27)	1.21 (0.81, 1.80)	1.04 (0.70, 1.55)	0.78 (0.49, 1.23)
Southwest	**1.97 (1.67, 2.32)**	**0.45 (0.36, 0.56)**	0.88 (0.77, 1.01)	1.08 (0.96, 1.22)	**0.74 (0.66, 0.84)**	1.10 (0.96, 1.26)
Adjusted^e^						
Alaska	**6.94 (5.42, 8.89)**	**1.77 (1.25, 2.50)**	**0.67 (0.58, 0.77)**	**1.20 (1.04, 1.40)**	0.90 (0.78, 1.03)	0.90 (0.77, 1.04)
East	**1.98 (1.49, 2.64)**	0.78 (0.50, 1.21)	**0.70 (0.56, 0.86)**	1.01 (0.81, 1.27)	0.86 (0.71, 1.05)	1.11 (0.89, 1.37)
Northern Plains	1.16 (0.71, 1.89)	1.06 (0.41, 2.77)	0.75 (0.49, 1.16)	1.28 (0.82, 2.00)	1.07 (0.71, 1.61)	0.80 (0.50, 1.27)
Southwest	**2.30 (1.94, 2.73)**	**0.46 (0.36, 0.59)**	0.90 (0.78, 1.04)	**0.46 (0.36, 0.59)**	**0.73 (0.64, 0.83)**	1.06 (0.92, 1.22)

^a^Pacific Coast was the region of reference for all analyses. No event was the reference group for all outcomes. Statistically significant associations are given in bold

^b^Patients underwent at least one documented treatment, including chemotherapy, radiation, or cancer-directed surgery. A total of 6775 individuals were included in this analysis

^c^Analysis included only patients that underwent cancer-directed surgery. A total of 6,493 individuals were included in this analysis. The reference category here was mastectomy with or without reconstruction/bi-lateral mastectomy with or without reconstruction

^d^Late stage was defined as SEER summary stage, regional or distant

^e^The model was adjusted for covariates including Diagnosis Year, Age Group, Ethnicity, Marital Status at Diagnosis, stage (except for the model where the outcome was diagnosis at late stage), and PRCDA

### Demographic and clinical characteristics: lung cancers

Demographic and clinical characteristics of lung cancer patients (*n* = 5,403) are given in Table 2. Mean age at diagnosis varied from 66.6 years (±10.9) in the East, to 69.6 years (±12.2) in the Southwest. Stage distribution also varied by region, with diagnosis at distant stage ranging from 50.7% in the East region to 63.5% in the Northern Plains region.

### Differences in components of care received by region, lung cancers

Frequencies and proportions of patients with lung cancer receiving each component of care by region are shown in Fig. 1b and given in Supplementary Table 7. Time to treatment ranged from a median of 21 days (IQR: 9, 32.5) in the Northern Plains, to 31 days (IQR: 11, 58) in the Pacific Coast region. The proportion of patients receiving treatment within 60 days also varied by region, from 63.3% in the Pacific Coast region, to 80.1% in the East. Receipt of cancer directed surgery varied from 13.1% in Alaska, to 20.9% in the East. The proportion of patients with surgery recommended but not performed varied from 2.8% in the Northern Plains, to 22.9% in the Southwest. Receipt of radiation varied from 27.6% in the Southwest, to 45.8% in Alaska; receipt of chemotherapy varied from 37.5% in the Southwest, to 47.7% in the Northern Plains.

Unadjusted and adjusted associations of region with component of care are given in Table 5; a forest plot showing the primary, multivariable-adjusted associations is given in Fig. 2b. Full results of the adjusted models are given in Supplementary Table 8. After adjustment for age at diagnosis, diagnosis year, PRCDA, ethnicity, marital status, and stage, region was significantly associated with time to treatment <=60 days, with patients in Alaska (OR 2.01 [1.68, 2.40]), the East (OR 2.33 [1.73, 3.13]), and the Southwest (OR 1.71 [1.27, 2.28]) having higher odds of timely treatment than those in the Pacific Coast. Patients in Alaska (OR 1.40 [1.16, 1.69]) and the Northern Plains (OR 2.35 [1.17, 4.70]) were more likely to be diagnosed at a late stage than those in the Pacific Coast. Patients in Alaska were also more likely to receive radiation (OR 1.42 [1.25, 1.62]) and less likely to receive surgery (OR 0.57 [0.46, 0.71]) than those in the Pacific Coast. Those in the Southwest were less likely to receive radiation (OR 0.64 [0.52, 0.80]) than those in the Pacific Coast.

**Table 5:** Unadjusted and multivariable-adjusted associations of region with components of treatment received for lung cancer among AIAN people; Surveillance, Epidemiology and Results program, 2000–2021, *n* = 5,403

Region^a^	Treatment Time^b^ (< = 60 days)	Microscopic confirmation	Surgery	Chemotherapy	Radiation	Diagnosed at late stage^c^
Unadjusted						
Alaska	**2.01 (1.68, 2.40)**	0.94 (0.78, 1.14)	**0.57 (0.46, 0.71)**	1.08 (0.95, 1.23)	**1.42 (1.25, 1.62)**	**1.40 (1.16, 1.69)**
East	**2.33 (1.73, 3.13)**	1.16 (0.84, 1.60)	1.24 (0.95, 1.63)	1.12 (0.91, 1.36)	1.00 (0.81, 1.22)	0.92 (0.71, 1.21)
Northern Plains	1.60 (0.95, 2.68)	1.05 (0.57, 1.93)	1.07 (0.62, 1.84)	1.41 (0.96, 2.08)	0.93 (0.62, 1.38)	**2.35 (1.17, 4.70)**
Southwest	**1.71 (1.27, 2.28)**	1.15 (0.84, 1.59)	**0.62 (0.44, 0.86)**	0.93 (0.76, 1.14)	**0.64 (0.52, 0.80)**	0.95 (0.73, 1.25)
Adjusted^d^						
Alaska	**2.25 (1.85, 2.74)**	0.87 (0.70, 1.09)	**0.70 (0.56, 0.87)**	1.01 (0.87, 1.17)	**1.43 (1.24, 1.64)**	**1.32 (1.08, 1.62)**
East	**1.86 (1.34, 2.59)**	1.04 (0.72, 1.52)	0.87 (0.63, 1.19)	1.26 (0.98, 1.61)	1.16 (0.92, 1.47)	0.95 (0.71, 1.28)
Northern Plains	1.19 (0.69, 2.04)	0.95 (0.50, 1.81)	1.18 (0.65, 2.12)	1.33 (0.88, 2.02)	1.00 (0.66, 1.52)	**2.44 (1.21, 4.94)**
Southwest	**1.75 (1.29, 2.36)**	1.37 (0.97, 1.93)	0.76 (0.55, 1.05)	1.02 (0.82, 1.28)	**0.66 (0.53, 0.82)**	0.94 (0.71, 1.24)

^a^Pacific Coast was the region of reference for all analyses. No event was the reference group for all outcomes. Statistically significant associations are given in bold

^b^Patients underwent at least one documented treatment, including chemotherapy, radiation, or cancer-directed surgery. A total of 3,671 individuals were included

^c^SEER derived summary stage from 2004 onwards. A total of 4,811 individuals were included. Late stage was defined as SEER summary stage, regional or distant

^d^The model was adjusted for covariates including Diagnosis Year, Age Group, Ethnicity, Sex, Marital Status at Diagnosis, stage (except for the model where the outcome was diagnosis at late stage), and PRCDA

### Demographic and clinical characteristics: colorectal cancers

Demographic and clinical characteristics of colorectal cancer patients (*n* = 5,626) are given in Table 3. Mean age at diagnosis varied from 61.2 years (±14.3) in the Southwest, to 64.8 years (±13.1) in the Northern Plains. Stage distribution also varied by region, with diagnosis at distant stage ranging from 18.1% in the Northern Plains to 26.2% in the East. Among those tested, the proportion of KRAS abnormalities and MSI unstable cancers varied by region (we were unable to compare KRAS results for the East and Northern Plains, and MSI results in the Northern Plains, due to low numbers).

### Differences in components of care received by region, colorectal cancers

Frequencies and proportions of patients with colorectal cancer receiving each component of care by region are shown in Fig. 1c and given in Supplementary Table 9. Median time to treatment varied by region, ranging from 8 days (0, 30) in the East to 15 days (0, 36) in the Pacific Coast. The proportion of patients receiving treatment within 60 days ranged from 69% in the Northern Plains, to 95% in Alaska. The proportion of colon cancer patients receiving partial or total colectomy, and the proportion of rectal cancer patients receiving total proctectomy, varied by region. KRAS testing documentation among stage IV cases varied from 34.8% in the East to 56.2% in Alaska (Northern Plains excluded due to small numbers). Documentation of MSI status ranged from 47.0% in the Southwest to 61.9% in the Northern Plains. The proportion of patients with 12+ regional nodes examined varied from 43.8% in Alaska to 53.0% in the East.

Unadjusted and adjusted associations of region with component of care are given in Table 6; a forest plot showing the primary, multivariable-adjusted associations is given in Fig. 2c. Full results of the adjusted models are given in Supplementary Table 10. Unadjusted and multivariable adjusted associations of region with component of care, stratified by cancer site are given in Supplementary Tables 11 (Colon only) and 12 (Rectum only). KRAS testing documentation was not included in these analyses due to the small numbers. After adjustment for age at diagnosis, diagnosis year, PRCDA, ethnicity, marital status, and stage, region was significantly associated with time to treatment <=60 days, with patients in Alaska (OR 4.80 [3.42, 6.73]), the East (OR 2.45 [1.47, 4.09]) and the Southwest (OR 2.20 [1.67, 2.89]) having higher odds of timely treatment than those in the Pacific Coast. Compared to the Pacific Coast, patients in Alaska had lower odds of 12+ regional nodes being examined (OR 0.69 [0.61, 0.79]) and MSI documentation (OR 0.74 [0.57, 0.98]); and, patients in the Southwest had lower odds of 12+ regional nodes being examined (OR 0.71 [0.62, 0.83], MSI testing documentation (OR 0.65 [0.50, 0.84], and receipt of surgery for either colon or rectal cancer.

**Table 6:** Unadjusted and multivariable-adjusted associations of region with components of treatment received for colorectal cancer among AIAN people; Surveillance, Epidemiology and Results program, 2000–2021, *n* = 5,626

Region^a^	Time to Treatment within 60 days^b^	Microscopic Confirmation	Regional Nodes 12 examined	MSI testing documented^c^	Receipt of Surgery, Colon only^d^	Receipt of Surgery, Rectum only^d^	**Chemotherapy**	**Radiation**	**Diagnosed at** **late stage**^e^
Unadjusted									
Alaska	**4.80 (3.42, 6.73)**	0.96 (0.63, 1.47)	**0.69 (0.61, 0.79)**	**0.74 (0.57, 0.98)**	**1.27 (1.02, 1.59)**	1.24 (0.95, 1.62)	0.89 (0.78, 1.02)	1.13 (0.95, 1.34)	0.96 (0.83, 1.11)
East	**2.45 (1.47, 4.09)**	0.63 (0.34, 1.17)	1.00 (0.80, 1.26)	0.78 (0.51, 1.19)	0.78 (0.55, 1.11)	1.03 (0.66, 1.60)	0.88 (0.70, 1.11)	0.88 (0.64, 1.20)	1.12 (0.87, 1.43)
Northern Plains	0.53 (0.27, 1.05)	1.90 (0.26, 13.93)	0.81 (0.52, 1.26)	1.19 (0.48, 2.90)	1.31 (0.55, 3.13)	1.14 (0.52, 2.46)	1.14 (0.73, 1.78)	1.45 (0.85, 2.48)	0.78 (0.48, 1.26)
Southwest	**2.20 (1.67, 2.89)**	0.90 (0.59, 1.38)	**0.71 (0.62, 0.82)**	**0.65 (0.50, 0.84)**	**0.71 (0.57, 0.87)**	**0.77 (0.60, 0.99)**	1.05 (0.92, 1.20)	1.08 (0.90, 1.28)	1.03 (0.89, 1.19)
Adjusted^f^									
Alaska	**4.98 (3.45, 7.17)**	0.86 (0.53, 1.38)	**0.70 (0.60, 0.82)**	**0.69 (0.51, 0.95)**	1.17 (0.86, 1.58)	1.30 (0.93, 1.83)	0.99 (0.84, 1.18)	**1.34 (1.10, 1.62)**	1.03 (0.88, 1.20)
East	**2.06 (1.19, 3.59)**	0.58 (0.28, 1.20)	0.95 (0.73, 1.24)	0.72 (0.44, 1.17)	1.00 (0.63, 1.60)	1.25 (0.72, 2.17)	0.80 (0.60, 1.07)	0.80 (0.56, 1.13)	1.22 (0.93, 1.59)
Northern Plains	0.54 (0.26, 1.09)	1.62 (0.21, 12.74)	0.76 (0.47, 1.23)	1.74 (0.66, 4.60)	1.52 (0.52, 4.49)	1.12 (0.46, 2.75)	1.38 (0.81, 2.36)	1.52 (0.87, 2.67)	0.77 (0.47, 1.27)
Southwest	**2.26 (1.70, 3.01)**	1.00 (0.62, 1.60)	**0.68 (0.59, 0.79)**	**0.64 (0.48, 0.85)**	**0.65 (0.49, 0.86)**	**0.72 (0.54, 0.98)**	1.01 (0.86, 1.19)	1.09 (0.90, 1.32)	1.04 (0.89, 1.21)

^a^The Pacific Coast was the region of reference for all analyses. No event was the reference group for all outcomes. Statistically significant associations are given in bold

^b^Patients underwent at least one documented treatment, including chemotherapy, radiation, cancer-directed surgery. A total of 5006 individuals were included

^c^MSI testing from 2018 onwards. A total of 1375 individuals were included in MSI analyses

^d^A total of 3657 individuals were included in analyses of Receipt of Surgery-Colon, and a total of 1859 individuals were included in analyses of Receipt of Surgery-Rectum

^e^SEER derived summary stage from 2004 onwards. A total of 5,174 participants were included. Late stage was defined as SEER summary stage, regional or distant

^f^The model was adjusted for covariates, including Diagnosis Year, Age Group, Sex, Ethnicity, Marital Status at Diagnosis, stage (except for the model where the outcome was diagnosis at late stage), and PRCDA

### Differences between AIAN people and NHW

To contextualize the primary findings within known disparities in cancer incidence and outcomes between AIAN people and NHW, we also compared treatments received for each of the three cancers studied herein between these populations. Characteristics of matched AIAN and NHW individuals are given in Supplementary Table 13 for female breast cancers, Supplementary Table 14 for lung cancers, and Supplementary Table 15 for colorectal cancers. Frequencies of individuals receiving components of care among matched AIAN and NHW people are given in Supplementary Table 16 for female breast cancers, Supplementary Table 17 for lung cancers, and Supplementary Table 18 for colorectal cancers. Finally, unadjusted and multivariable adjusted associations of race with components of treatment received are given in Supplementary Table 19 for female breast cancers, Supplementary Table 20 for lung cancers, and Supplementary Table 21 for colorectal cancers. Among female breast cancer patients, AIAN people were less likely to receive treatment within 60 days (OR 0.53 [0.46, 0.62]), less likely to receive radiation (OR 0.73 [0.65, 0.81]), and less likely to receive breast conserving surgery (OR 0.81 [0.72, 0.91]). AIAN women were also more likely to be diagnosed with late-stage breast cancer (OR 1.44 [1.30, 1.61]). Among lung cancer patients, AIAN people were less likely to receive treatment within 60 days (OR 0.73 [0.63, 0.84]), receive microscopic confirmation (OR 0.63 [0.53, 0.74]), chemotherapy (OR 0.83 [0.75,0.91]), radiation (OR 0.88 [0.80,0.97]), or surgery (OR 0.70 [0.61, 0.80]). AIAN people were more likely to be diagnosed at a later stage than NHW (OR 1.19 [1.05, 1.34]). Among colorectal cancer patients, AIAN people were less likely to receive treatment within 60 days (OR 0.50[0.44, 0.57]), receive microscopic confirmation (OR 0.60 [0.41,0.87]), surgery for treatment of colon cancer (OR 0.80 [0.65,0.97]), have 12+ regional nodes examined (OR 0.83 [0.77,0.91]), or MSI testing documented (OR 0.72 [0.60,0.87]). AIAN people were more likely to be diagnosed at a late stage (OR 1.21 [1.10, 1.32]) and to receive radiation (OR 1.25 [1.12, 1.39]).

## Discussion

In this study, we report regional differences in diagnostic and treatment-related metrics for female breast, lung, and colorectal cancers diagnosed among AIAN patients in the U.S., using population-based data from the SEER17 database. We found regional differences in treatments received, as well as metrics known to indicate care quality, including time to treatment (all sites examined), ER/PR/HER2 testing (female breast cancers only), 12+ regional lymph nodes examined, KRAS and MSI testing (colorectal cancers only). Several studies have indicated regional differences in cancer survival among AIAN peoples [1, 10]; yet, reasons for these differences are not well understood. Regional differences in survival likely reflect a combination of factors, including cancer biology, access to and quality of cancer and end of life care; and other social and structural factors and individual behaviors [22–24]. Indeed, a recent study by our group using SEER-Medicare data showed that predictors of breast, colorectal, and lung cancer death among AIAN patients include IHS region, age, stage, comorbidities, marital status, and area-based social measures [25]. While we did not directly assess associations with survival in this study, our results provide compelling evidence to suggest that regional differences in treatments received and quality of care *may* contribute to regional differences in cancer survival among AIAN people, a hypothesis that warrants further investigation. Critically, in our secondary analysis comparing AIAN people to NHW, we also observed differences in treatments received and lower adherence to care quality metrics among AIAN people. Cancer survival differences between AIAN people and NHW have been well demonstrated [1, 10]; yet, when AIAN people receive the same guideline concordant treatment as their NHW counterparts, these disparities diminish [6]. Thus, while our study provides further evidence to support the need for high quality cancer care across the Tribal health system to address AIAN cancer disparities compared to NHW, it also adds novel findings regarding the need for equitable access to high-quality care across IHS regions.

While there is a plethora of research examining regional differences in cancer incidence and mortality among AIAN people [1, 3, 4, 18, 20, 26–28], scant research has examined regional differences in treatments. To our knowledge, only one study has addressed this issue: Erdrich and colleagues examined regional variations in breast conserving treatment for AIAN women and found that receipt of lumpectomy varied from 51.1% in the Northern Plains to 64.2% in the East [29]. These findings differ slightly to those presented herein, where we report variations in lumpectomy from 51.2% in the East, to 54.3% in the Northern Plains. These diverse findings could be explained by differences in the data source (the prior study used data from U.S. Cancer Statistics, which includes all 50 U.S. states, whereas we used SEER data to ensure access to a wider range of outcome variables), and/or in the study population (the prior study restricted to patients living in PRCDA counties at the time of diagnosis, whereas we included all AIAN people regardless of PRCDA residence, and adjusted for PRCDA residence in our multivariable models). Further, the present study included a far longer time-period than that examined by Erdrich and colleagues. Regional differences in treatments received may reflect differences in practice patterns, and/or access to care; unfortunately, the data presented herein cannot explain why we observe regional differences, just that these differences exist. Clinical characteristics of the tumors may also contribute; however, we did adjust both for stage at diagnosis (all cancer sites) and breast cancer subtype (breast cancer only) in our multivariable adjusted models. Further research is required to understand why regional patterns in cancer treatment exist among AIAN people, and whether these regional differences may mediate observed associations of region with cancer survival [10].

While we did observe some regional differences in treatments received (e.g., use of radiation therapy for lung and colorectal cancers) that may reflect regional differences in practice patterns, differences in quality-of-care metrics may be more urgent to explain, given that most (if not all) patients should be receiving these procedures as part of their diagnostic and staging process. Indeed, quality metrics are defined because adherence is known to improve survival [30]. In our study, we observed regional differences in diagnostic quality of care-related measures, including ER/PR/HER2 testing for female breast cancers, and 12+ regional lymph nodes examined for colorectal cancers. ER/PR/HER2 testing should be conducted for all breast cancers, to determine the best course of treatment [31]. Similarly, examination of 12+ regional lymph nodes for colorectal cancers is associated with improved staging and survival [32], and is therefore a quality of care metric employed by both the National Comprehensive Cancer Network [33, 34] and the Commission on Cancer [35]. Differences in these metrics are troubling, given that all AIAN patients should be receiving care that is adherent to these standards.

We also observed differences in timely care for all cancers. Timely care is considered a hallmark of quality care, and a predictor of cancer survival [36, 37]. In adjusted models, Alaska consistently exhibited shorter median time to care and had substantially higher odds of treatment in <=60 days, relative both to the reference group (Pacific Coast) and all other regions. These findings may initially appear surprising, because Alaska’s geography holds unique challenges for cancer care provision. Approximately 86% of Alaska Native (AN) people live off the road system [38], resulting in heavy reliance on aviation for healthcare transportation. Healthcare is provided to the majority of AN cancer patients in Anchorage through a “spoke and hub” model of care that requires extensive travel for patients living outside this area [39]. However, one key difference between Alaska and other IHS regions with regards to provision of cancer care is that the Alaska Tribal Health System is 100% Tribally owned and operated, with IHS funds compacted (i.e., Public Law 93–638 Title V compacting, versus Title I contracting) to provide healthcare across 13 different Tribal healthcare organizations [39]. All cancer treatment within this system is provided by the Alaska Native Tribal Health Consortium at the Alaska Native Medical Center in Anchorage. In contrast, Tribal cancer care outside of Alaska may be provided directly by the IHS, or by Tribally managed healthcare facilities that are either contracted (Title I contracting) or compacted to provide healthcare to their people. Critically, access to Tribally-managed cancer care in the contiguous U.S. is limited, meaning that most AIAN patients receiving cancer care through the IHS must be referred to purchased/referred care outside of the Tribal health system [1], a complicated process which may result in treatment delays. In contrast, the Alaska Tribal Health System may provide more efficient and timely referrals for cancer care, since there is rarely a need to refer outside of the system. Alternatively, since all the cancers examined herein are screenable, patients traveling from rural Alaska to Anchorage for screening may be routed directly from diagnosis to treatment, resulting in very short time to treatment. This hypothesis is supported by one study examining time from screening to treatment among AN breast cancer patients [40]. Patients in the contiguous U.S. may also experience different barriers to accessing care in a timely manner, including long travel distances to treatment by car, and/or seasonal weather that impacts car travel. While further research is necessary to understand why we observe such distinct regional variation in timely treatment, there may be opportunities for Tribal and healthcare leaders across regions to share best practices for timely cancer care and lessons learned to improve healthcare for all AIAN people.

This study has limitations that must be considered. First, due to the use of the SEER dataset, we were constrained in the outcomes and covariates available for analysis. For example, we were unable to assess regional variation in adherence to guideline-recommended treatment or adjust for potentially important confounders such as the number and type of comorbidities. A key dataset often used to address these limitations is the SEER-Medicare linked database; however, given the limited participation in this database and its inclusion of only those aged 65 years or older, small sample sizes may preclude the ability to conduct regional analyses. Even the SEER public-use dataset used herein is limited in its reach, representing about 33% of AIAN people [41], and lacks data on the Southern Plains, with limited representation in the Northern Plains and the East. Thus, our data may not be representative of each of the regions presented herein. National datasets such as the North American Association for Central Cancer Registries (NAACCR) Cancer in North America (CiNA) dataset, or the U.S. Cancer Statistics database include data on all 50 states, but these databases do not include the extended suite of treatment variables presented herein. Critically, the potential accuracy of treatment variables within SEER should be considered when interpreting these results. Chemotherapy is particularly problematic: an analysis comparing SEER and SEER-Medicare linked data indicated that the sensitivity of the chemotherapy variable in SEER was as low as 68% [16]. Further, radiotherapy data may also be incomplete, with a sensitivity in the aforementioned paper of 80% [16]. Whilst sensitivity of SEER cancer treatment information has never been evaluated for AIAN people, we encourage interpretation of this information with caution. While we consider it unlikely that the accuracy/completeness of treatment variables should differ by IHS region, or race, meaning relative comparisons between groups would remain valid, this assumption has not been formally tested. Additionally, while we perceive the inclusion of all AIAN people in this analysis to be a strength (as many other analyses exclude non-PRCDA residents and Hispanic AIAN people), we do recognize that there is the potential for racial misclassification, such that we may be missing AIAN people who should be included in this analysis; this misclassification is likely to vary by residence in a PRCDA county [17, 42]. While all cancer registries routinely link with the HIS [17, 42], and additional methods are being developed to address racial misclassification at the local, regional, and national levels [43], methods such as linkage to local Tribal rolls have not yet been implemented into routine surveillance practice. We did not adjust for multiple comparisons in this study; therefore, it is possible that some of the associations presented herein may be spurious. We made this decision weighing the consequences of making a type II error versus a type I error, given that this is the first study on this topic, and results pertain to an underserved, minoritized population [44, 45]. Finally, we examined treatments received over a long-time period, over which it is expected that practice patterns and available treatments would have changed; while we did adjust for year of diagnosis in all models, we may not have fully accounted for secular trends.

## Conclusions

This is one of the first studies to examine regional differences in cancer treatment and related care-quality metrics among AIAN people. These data are an important first step toward understanding why we might observe regional variation in cancer survival among AIAN people [1, 10]. Further research is needed to understand any potential link between regional differences in access to and receipt of care and cancer outcomes, as well as the interplay with other clinical, social, and structural factors; yet, currently available datasets do not provide the detailed data necessary to answer these questions while retaining sample sizes sufficient to make regional comparisons. Local efforts to understand cancer care patterns may be needed to extend the initial work presented herein to provide data to support interventions needed to address the high and varying burden of cancer among AIAN people.

## Supplementary Information

Below is the link to the electronic supplementary material.

Supplementary file1 (DOCX 169 KB)

## Data Availability

Data are available through application to the NCI’s SEER Program.

## Abbreviations

AIAN: American Indian and Alaska native
PRCDA: Purchased/referred care deliver areas
SEER: Surveillance, epidemiology and end results
