# Supplementary material for: Regional differences in cancer treatment among American Indian and Alaska native peoples: a surveillance, epidemiology, and end results study
Source: Cancer Causes Control. 2026 Nov 3;37(11):187. doi: 10.1007/s10552-026-02271-9 (PMC13731451; doi:10.1007/s10552-026-02271-9)
Supplement: Supplementary file 1 [file 10552_2026_2271_MOESM1_ESM.docx]

**Regional Differences in Cancer Treatment among American Indian and Alaska Native Peoples: A Surveillance, Epidemiology, and End Results Study**

Faith Elenwa,^1^ Jennifer Erdrich,^2^ Jamie Sorensen,^1^ Vanessa E Slater,^1,3^ Carol Goldtooth^7^, Priscilla R. Sanderson^7,8^, Tamee Livermont,^9^ Keely Ulmer,^4,5^ Bradley D McDowell,^4^ Sarah H Nash,^1,4,6^

^1^Department of Epidemiology, College of Public Health, University of Iowa, Iowa City IA

^2^Department of Surgery, University of Arizona, Tucson AZ

^3^Department of Epidemiology, Gillings School of Global Public Health, University of North Carolina, Chapel Hill NC

^4^Holden Comprehensive Cancer Center, University of Iowa, Iowa City IA

^5^Monument Health, Rapid City SD

^6^University of New Mexico Comprehensive Cancer Center, University of New Mexico, Albuquerque NM

^7^Partnership for Native American Cancer Prevention, Northern Arizona University

^8^Health Sciences Department, College of Health and Human Services, Northern Arizona University

^9^University of California San Francisco East Bay General Surgery Residency

**Corresponding Author:** Sarah Nash. Current address: Cancer Research Facility (CRF), Bldg 229, 1 University of New Mexico, MSC11 6020, Albuquerque, NM 87131-0001. Email: [SarNash@salud.unm.edu](mailto:SarNash@salud.unm.edu)

**Journal:** Cancer Causes & Control

**Supplementary Table 1.** Details on variable names and strata definitions used for the analyses presented herein.

| **Variable** | **SEER variable name** | **Cancer site** | **Strata definitions** |
| --- | --- | --- | --- |
| *Exposure(s)* | | | |
| IHS Region | SEER_Registry | Breast, Lung, Colorectal | East (Kentucky, Georgia, Connecticut, New Jersey, and Louisiana), Northern Plains (Iowa), Southwest (New Mexico, Utah), Pacific Coast (California, Hawaii, Seattle), and Alaska (Alaska Native Tumor Registry) |
| Race | Race_recode_W_B_AI_API | Breast, Lung, Colorectal | White, American Indian and Alaska Native |
| *Outcomes* | | | |
| Time to treatment initiation | Time From Diagnosis to Treatment In Days_Recode | Breast, Lung, Colorectal | Numeric variable, stratified as <= and >60 days |
| Microscopic confirmation | Diagnostic_Confirmation | Breast, Lung, Colorectal | Yes (Positive exfoliative; Positive histology; Positive microscopic) and No (Clinical diagnosis only; Radiography without microscopic confirmation; Unknown) |
| Receipt of surgery | Rx_Summ_Surg_Primary Site | Breast, Lung, Colorectal | Yes (All surgery-related codes), No (00), and Unknown (90,99) |
| Reason for no cancer directed surgery | No_Cancer_Surgery | Breast, Lung, Colorectal | Surgery performed; Not recommended; Recommended but not performed (Recommended but not performed, unknown reason; recommended but not performed, patient refused, recommended, unknown if performed); Unknown (Unknown; death certificate only case) |
| Receipt of radiation | Rx_Summ_Radiation | Breast, Lung, Colorectal | Yes (Beam radiation; Combination of beam with implants or isotopes; Radiation, NOS method or source not specified; Radioactive implants [includes brachytherapy]; Radioisotopes) and No (None/Unknown; Recommended, unknown if administered; Refused) |
| Receipt of chemotherapy | Rx_Summ_Chemotherapy | Breast, Lung, Colorectal | Yes (Yes) and No (No, Unknown) |
| Late stage diagnosis | Combined_Stage | Breast, Lung, Colorectal | Yes (Regional, Distant) and No (Local, Unknown) |
| Receipt of breast conserving surgery, among those who received surgery | Rx_Summ_Surg_Primary Site | Breast | Yes (19-24) and No, including mastectomy and bilateral mastectomy with or without reconstruction (40, 41, 50, 51, 53, 54, 55, 56, 60, 61, 70, 71, 80, 30, 43-46, 52, 57, 58, 59, 63, 64, 65, 66, 67, 42, 76, 62, 72, 47 - 49, 75, 68, 69, 73, 74) |
| Receipt of definitive ER/PR testing | ER_Status_Recode and PR_Status_Recode | Breast | Yes (Positive or Negative on BOTH variables) and No (Borderline or Unknown on EITHER variable) |
| Receipt of definitive HER2 testing | Derived_HER2_Recode | Breast | Yes (Positive, Negative) and No (Borderline, Unknown) |
| Most extensive surgery: colon cancer | Rx_Summ_Surg_Primary Site | Colon | None (00), Minor surgery (10, 12, 20, 22, 24, 26, 27, 28, 29), Partial colectomy (30, 31, 32), Subtotal or total colectomy (40, 41, 50, 51, 60, 61, 70, 80) |
| Most extensive surgery: rectal cancer | Rx_Summ_Surg_Primary Site | Rectum | None (00), Minor surgery (10, 12, 20, 22, 24, 26, 27, 28, 29), Wedge or segmental resection; pull through with sphincter preservation (30, 31, 40), Total proctectomy or proctocolectomy (50, 51, 55, 60, 70, 80) |
| KRAS testing documented | KRAS | Colorectal | Yes (Abnormal; normal (wild type)), No (Not documented, KRAS not assessed; Test ordered, results not in chart) |
| Microsatellite Instability (MSI) testing documented | MSI | Colorectal | Yes (MSI Stable, MSI Unstable) and No (Not documented; MSI-indeterminate; Not assessed or unknown if assessed) |
| 12+ Regional lymph nodes examined | Regional_Lymph_Nodes_  Examined |  | Yes (>=12) and No (<=11) |
| *Covariates* | | | |
| Age | Age_1yr | Breast, Lung, Colorectal | 20-39 years, 40-64 years, and 65+ years |
| Ethnicity | Origin Recode NHIA (NAACCR Hispanic Identification Algorithm) | Breast, Lung, Colorectal | Hispanic, non-Hispanic |
| Stage | Combined_Stage | Breast, Lung, Colorectal | Local, Regional, Distant, and Unknown |
| Marital status |  | Breast, Lung, Colorectal | Partnered (Married, Unmarried or domestic partner), unpartnered (Divorced, Widowed, Separated; Single or never married), or unknown |
| Rurality | Rural_Urban_Code | Breast, Lung, Colorectal | Urban (Counties in metropolitan areas greater than or equal to 1 million population, Counties in metropolitan areas of 250,000 to 1 million, Counties in metropolitan areas of less than 250 thousand) and rural (all other categories) |
| Diagnosis year |  | Breast, Lung, Colorectal |  |
| Income | Median_Household_income inflation adjusted | Breast, Lung, Colorectal | <$40,000, $40,000-$100,000 (All categories between $40,000 and $100,000), and >$100,000 |
| PRCDA | PRCDA 2020 | Breast, Lung, Colorectal |  |
| CoC Accredited | CoCAccredited_flag | Breast, Lung, Colorectal | Yes (Analytic abstract) and No (Abstract from non-CoC; non-Analytic abstract) |

**Supplementary Table 2.** Characteristics of AIAN people with breast cancer (females only), stratified by IHS region, among the subset of individuals with subtype information. Data from the Surveillance, Epidemiology, and End Results program, 2010-2021, n = 4,647.

| **Category** | **Alaska**  **(N=796)** | **East**  **(N=379)** | **Northern Plains**  **(N=67)** | **Pacific Coast**  **(N=2481)** | **Southwest**  **(N=924)** |
| --- | --- | --- | --- | --- | --- |
| Diagnosis Age (mean (SD)) | | | | | |
|  | 58.7 (12.8) | 59.1 (14) | 59.4 (11.6) | 60.1 (12.8) | 58.9 (13.1) |
| Age Group (%) | | | | | |
| 20-39 | 52 (6.5%) | 24 (6.3%) | <5 | 146 (5.9%) | 69 (7.5%) |
| 40-64 | 487 (61.2%) | 216 (57%) | 42 (62.7%) | 1365 (55%) | 550 (59.5%) |
| 65+ | 257 (32.3%) | 139 (36.7%) | 23 (34.3%) | 970 (39.1%) | 305 (33%) |
| Income (%) | | | | | |
| < $40,000 | 0 (0%) | 6 (1.6%) | 0 (0%) | 0 (0%) | 152 (16.5%) |
| $40,000-100,000 | 796 (100%) | 325 (85.8%) | 67 (100%) | 2093 (84.4%) | 769 (83.2%) |
| >= $100,000 | 0 (0%) | 48 (12.7%) | 0 (0%) | 388 (15.6%) | <5 |
| Ethnicity (%) | | | | | |
| Non-Hispanic | 795 (99.9%) | 343 (90.5%) | 65 (97%) | 2228 (89.8%) | 849 (91.9%) |
| Hispanic | <5 | 36 (9.5%) | <5 | 253 (10.2%) | 75 (8.1%) |
| Marital status at diagnosis (%) | | | | | |
| Not Partnered | 270 (33.9%) | 159 (42%) | 30 (44.8%) | 1130 (45.5%) | 430 (46.5%) |
| Partnered | 363 (45.6%) | 201 (53%) | 31 (46.3%) | 1182 (47.6%) | 341 (36.9%) |
| Unknown | 163 (20.5%) | 19 (5%) | 6 (9%) | 169 (6.8%) | 153 (16.6%) |
| RUCC (%) | | | | | |
| Rural | 796 (100%) | 52 (13.7%) | 30 (44.8%) | 334 (13.5%) | 370 (40%) |
| Urban | 0 (0%) | 327 (86.3%) | 37 (55.2%) | 2147 (86.5%) | 554 (60%) |
| Diagnosis Year (%) | | | | | |
| 2006-2010 | 58 (7.3%) | 29 (7.7%) | <5 | 173 (7%) | 64 (6.9%) |
| 2011-2015 | 339 (42.6%) | 117 (30.9%) | 27 (40.3%) | 952 (38.4%) | 367 (39.7%) |
| 2016-2021 | 399 (50.1%) | 233 (61.5%) | 38 (56.7%) | 1356 (54.7%) | 493 (53.4%) |
| PRCDA (%) | | | | | |
| Yes | 796 (100%) | 70 (18.5%) | 25 (37.3%) | 1784 (71.9%) | 902 (97.6%) |
| No | 0 (0%) | 309 (81.5%) | 42 (62.7%) | 697 (28.1%) | 22 (2.4%) |
| CoC Accredited Flag (%)^a^ | | | | | |
| Yes | 20 (7.1%) | 140 (84.3%) | 18 (75%) | 725 (76.4%) | 219 (65%) |
| No | 8 (2.8%) | 9 (5.4%) | 0 (0%) | 47 (5%) | 18 (5.3%) |
| Grade (%)^b^ | | | | | |
| Well differentiated | 218 (27.4%) | 75 (19.8%) | 17 (25.4%) | 528 (21.3%) | 171 (18.5%) |
| Moderately differentiated | 307 (38.6%) | 144 (38%) | 25 (37.3%) | 1018 (41%) | 317 (34.3%) |
| Poorly differentiated | 184 (23.1%) | 132 (34.8%) | 23 (34.3%) | 747 (30.1%) | 282 (30.5%) |
| Undifferentiated | 0 (0%) | <5 | 0 (0%) | 6 (0.2%) | 0 (0%) |
| Unknown | 87 (10.9%) | 27 (7.1%) | <5 | 182 (7.3%) | 154 (16.7%) |
| Stage (%) | | | | | |
| Localized | 502 (63.1%) | 228 (60.2%) | 46 (68.7%) | 1505 (60.7%) | 536 (58%) |
| Regional | 236 (29.6%) | 120 (31.7%) | 17 (25.4%) | 771 (31.1%) | 281 (30.4%) |
| Distant | 51 (6.4%) | 26 (6.9%) | <5 | 158 (6.4%) | 72 (7.8%) |
| Unknown/Unstaged | 7 (0.9%) | 5 (1.3%) | <5 | 47 (1.9%) | 35 (3.8%) |
| Breast cancer subtype (%) | | | | | |
| HR+/HER2+ | 99 (12.4%) | 37 (9.8%) | 8 (11.9%) | 253 (10.2%) | 115 (12.4%) |
| HR+/HER2- | 548 (68.8%) | 263 (69.4%) | 45 (67.2%) | 1709 (68.9%) | 563 (60.9%) |
| HR-/HER2+ | 45 (5.7%) | 19 (5%) | <5 | 114 (4.6%) | 42 (4.5%) |
| HR-/HER2- | 77 (9.7%) | 38 (10%) | 10 (14.9%) | 251 (10.1%) | 93 (10.1%) |
| No/Unknown | 27 (3.4%) | 22 (5.8%) | <5 | 154 (6.2%) | 111 (12%) |

^a^CoC Accredited Flag from 2018 onwards. A total of 1,204 individuals were included in this subset.

^b^From 2000 to 2017, variable Grade Recode was used to define grade; from 2018 to present, pathological grade was used.

**Supplementary Table 3**. Frequencies of individuals receiving components of care among AIAN people with breast cancer, females only, by region. Data from the Surveillance, Epidemiology, and End Results program, n=7,221.

| **Category** | **Alaska**  **(N=1340)** | **East**  **(N=532)** | **Northern Plains**  **(N=100)** | **Pacific Coast**  **(N=3820)** | **Southwest**  **(N=1429)** |
| --- | --- | --- | --- | --- | --- |
| Time to Treatment [IQR]^a^ | | | | | |
|  | 22 [13, 34] | 27 [13, 45] | 27 [14, 37] | 32 [17, 50] | 27 [11, 46] |
| Time to Treatment ≤60 days (%)^a^ | | | | | |
| Yes | 1198 (93.6%) | 433 (86.8%) | 72 (76.6%) | 2603 (72.3%) | 1089 (83.7%) |
| No | 68 (5.3%) | 60 (12%) | <5 | 556 (15.4%) | 203 (15.6%) |
| Unknown | 14 (1.1%) | 6 (1.2%) | 19 (20.2%) | 442 (12.3%) | 9 (0.7%) |
| Microscopic Confirmation (%) | | | | | |
| Yes | 1334 (99.6%) | 530 (99.6%) | 100 (100%) | 3794 (99.3%) | 1419 (99.3%) |
| No/Unknown | 6 (0.4%) | <5 | 0 (0%) | 26 (0.7%) | 10 (0.7%) |
| Chemotherapy (%)^b^ | | | | | |
| Yes | 618 (46.1%) | 243 (45.7%) | 48 (48%) | 1652 (43.2%) | 646 (45.2%) |
| No/Unknown | 722 (53.9%) | 289 (54.3%) | 52 (52%) | 2168 (56.8%) | 783 (54.8%) |
| Radiation (%)^b^ | | | | | |
| Yes | 652 (48.7%) | 250 (47%) | 52 (52%) | 1948 (51%) | 623 (43.6%) |
| No/Unknown | 688 (51.3%) | 282 (53%) | 48 (48%) | 1872 (49%) | 806 (56.4%) |
| Cancer-directed surgery (%) | | | | | |
| Surgery performed | 1242 (92.7%) | 475 (89.3%) | 92 (92%) | 3456 (90.5%) | 1239 (86.7%) |
| Not recommended | 61 (4.6%) | 45 (8.5%) | <5 | 296 (7.7%) | 111 (7.8%) |
| Recommended but not performed | 33 (2.5%) | 10 (1.9%) | <5 | 60 (1.6%) | 69 (4.8%) |
| Unknown | <5 | <5 | 0 (0%) | 8 (0.2%) | 10 (0.7%) |
| Receipt of surgery (%) | | | | | |
| Surgery received | 1240 (92.5%) | 473 (88.9%) | 92 (92%) | 3452 (90.4%) | 1236 (86.5%) |
| No/Unknown | 100 (7.5%) | 59 (11.1%) | 8 (8%) | 368 (9.6%) | 193 (13.5%) |
| Type of surgery (%) | | | | | |
| Mastectomy with or without reconstruction | 517 (41.7%) | 173 (36.6%) | 33 (35.9%) | 1060 (30.7%) | 460 (37.2%) |
| Lumpectomy, or other breast preserving surgery | 611 (49.3%) | 242 (51.2%) | 50 (54.3%) | 2024 (58.6%) | 687 (55.6%) |
| Bi-lateral Mastectomy with or without reconstruction | 112 (9%) | 58 (12.3%) | 9 (9.8%) | 368 (10.7%) | 89 (7.2%) |
| ER/PR testing occurred (%) | | | | | |
| Yes | 1285 (95.9%) | 502 (94.4%) | 95 (95%) | 3616 (94.7%) | 1270 (88.9%) |
| No/Unknown | 55 (4.1%) | 30 (5.6%) | 5 (5%) | 204 (5.3%) | 159 (11.1%) |
| Diagnosed at late stage (%)^c^ | | | | | |
| Yes | 408 (36.3%) | 191 (39.3%) | 28 (32.6%) | 1270 (38%) | 503 (39.8%) |
| No | 706 (62.9%) | 286 (58.8%) | 57 (66.3%) | 2005 (60%) | 721 (57%) |
| Unknown | 9 (0.8%) | 9 (1.9%) | <5 | 65 (1.9%) | 41 (3.2%) |

^a^ Patients underwent at least one documented treatment, including chemotherapy, radiation, or cancer-directed surgery. A total of 6,775 individuals were included in this analysis.

^b^Due to known issues of sensitivity in the chemotherapy and radiation variables (Noone et al. 2016), it is not recommended to interpret these data as reflecting prevalence of treatment receipt.

^c^SEER derived summary stage from 2004 onwards. A total of 6,300 individuals were included in this analysis.

**Supplementary Table 4**. Frequencies of individuals receiving components of care among AIAN females with breast cancer, among the subset of individuals with breast cancer subtype information, by region. Data from the Surveillance, Epidemiology, and End Results program, 2010-2021, n = 4,647.

| **Category** | **Alaska**  **(N=796)** | **East**  **(N=379)** | **Northern Plains**  **(N=67)** | **Pacific Coast**  **(N=2481)** | **Southwest**  **(N=924)** |
| --- | --- | --- | --- | --- | --- |
| Time to Treatment [IQR]^a^ | | | | | |
|  | 26 [16, 36] | 33 [17, 49] | 27 [15, 38] | 35 [21, 55] | 32 [20, 51] |
| Time to Treatment ≤60 days (%)^a^ | | | | | |
| Yes | 697 (92.7%) | 304 (85.6%) | 60 (93.8%) | 1775 (76.6%) | 665 (80.3%) |
| No | 47 (6.3%) | 46 (13%) | <5 | 468 (20.2%) | 160 (19.3%) |
| Unknown | 8 (1.1%) | 5 (1.4%) | <5 | 73 (3.2%) | <5 |
| Microscopic Confirmation (%) | | | | | |
| Yes | 792 (99.5%) | 379 (100%) | 67 (100%) | 2464 (99.3%) | 915 (99%) |
| No/Unknown | <5 | 0 (0%) | 0 (0%) | 17 (0.7%) | 9 (1%) |
| Chemotherapy (%)^b^ | | | | | |
| Yes | 366 (46%) | 166 (43.8%) | 30 (44.8%) | 1039 (41.9%) | 385 (41.7%) |
| No/Unknown | 430 (54%) | 213 (56.2%) | 37 (55.2%) | 1442 (58.1%) | 539 (58.3%) |
| Radiation (%)^b^ | | | | | |
| Yes | 424 (53.3%) | 182 (48%) | 38 (56.7%) | 1274 (51.4%) | 411 (44.5%) |
| No/Unknown | 372 (46.7%) | 197 (52%) | 29 (43.3%) | 1207 (48.6%) | 513 (55.5%) |
| Cancer-directed Surgery (%) | | | | | |
| Surgery performed | 719 (90.3%) | 334 (88.1%) | 63 (94%) | 2202 (88.8%) | 786 (85.1%) |
| Not recommended | 55 (6.9%) | 37 (9.8%) | <5 | 239 (9.6%) | 91 (9.8%) |
| Recommended but not performed | 20 (2.5%) | 7 (1.8%) | <5 | 37 (1.5%) | 37 (4%) |
| Unknown | <5 | <5 | 0 (0%) | <5 | 10 (1.1%) |
| Receipt of Surgery (%) | | | | | |
| Surgery received | 718 (90.2%) | 333 (87.9%) | 63 (94%) | 2199 (88.6%) | 785 (85%) |
| No/Unknown | 78 (9.8%) | 46 (12.1%) | <5 | 282 (11.4%) | 139 (15%) |
| Type of Surgery (%) | | | | | |
| Mastectomy with or without reconstruction | 244 (34%) | 114 (34.2%) | 20 (31.7%) | 606 (27.6%) | 252 (32.1%) |
| Lumpectomy, or other breast preserving surgery | 388 (54%) | 173 (52%) | 37 (58.7%) | 1298 (59%) | 467 (59.5%) |
| Bi-lateral Mastectomy with or without reconstruction | 86 (12%) | 46 (13.8%) | 6 (9.5%) | 295 (13.4%) | 66 (8.4%) |
| Breast cancer subtype known (%) | | | | | |
| Yes | 769 (96.6%) | 357 (94.2%) | 66 (98.5%) | 2327 (93.8%) | 813 (88%) |
| No | 27 (3.4%) | 22 (5.8%) | <5 | 154 (6.2%) | 111 (12%) |
| ER/PR testing occurred (%) | | | | | |
| Yes | 779 (97.9%) | 373 (98.4%) | 66 (98.5%) | 2405 (96.9%) | 844 (91.3%) |
| No/Unknown | 17 (2.1%) | 6 (1.6%) | <5 | 76 (3.1%) | 80 (8.7%) |
| HER2 testing occurred (%) | | | | | |
| Yes | 769 (96.6%) | 358 (94.5%) | 66 (98.5%) | 2332 (94%) | 816 (88.3%) |
| No/Unknown | 27 (3.4%) | 21 (5.5%) | <5 | 149 (6%) | 108 (11.7%) |
| Diagnosed at late stage (%) | | | | | |
| Yes | 287 (36.1%) | 146 (38.5%) | 20 (29.9%) | 929 (37.4%) | 353 (38.2%) |
| No | 502 (63.1%) | 228 (60.2%) | 46 (68.7%) | 1505 (60.7%) | 536 (58%) |
| Unknown | 7 (0.9%) | 5 (1.3%) | <5 | 47 (1.9%) | 35 (3.8%) |

^a^Patients underwent at least one documented treatment, including chemotherapy, radiation, or cancer-directed surgery. A total of 4,315 individuals were included in this analysis.

^b^Due to known issues of sensitivity in the chemotherapy and radiation variables (Noone et al. 2016), it is not recommended to interpret these data as reflecting prevalence of treatment receipt.

**Supplementary Table 5.** Full results of multivariable-adjusted models showing associations of region with components of treatment received for breast cancer among AIAN women. Data from the Surveillance, Epidemiology, and End Results program, 2000 – 2021, N=7,221.^a^

| **Characteristic** | **Level** | **Time to Treatment ≤ 60 days**^b^ | **ER PR testing occurred** | **Lumpectomy, or other breast-preserving surgery**^c^ | **Chemotherapy** | **Radiation** | **Diagnosed at late stage** |
| --- | --- | --- | --- | --- | --- | --- | --- |
| PRCDA Region (vs. Pacific Coast) | | | | | | | |
|  | Alaska | **6.94 (5.42, 8.89)** | **1.77 (1.25, 2.50)** | **0.67 (0.58, 0.77)** | **1.20 (1.04, 1.40)** | 0.90 (0.78, 1.03) | 0.90 (0.77, 1.04) |
|  | East | **1.98 (1.49, 2.64)** | 0.78 (0.50, 1.21) | **0.70 (0.56, 0.86)** | 1.01 (0.81, 1.27) | 0.86 (0.71, 1.05) | 1.11 (0.89, 1.37) |
|  | Northern Plains | 1.16 (0.71, 1.89) | 1.06 (0.41, 2.77) | 0.75 (0.49, 1.16) | 1.28 (0.82, 2.00) | 1.07 (0.71, 1.61) | 0.80 (0.50, 1.27) |
|  | Southwest | **2.30 (1.94, 2.73)** | **0.46 (0.36, 0.59)** | 0.90 (0.78, 1.04) | 1.08 (0.93, 1.24) | **0.73 (0.64, 0.83)** | 1.06 (0.92, 1.22) |
| Diagnosis Year (vs. 2000-2005) | | | | | | | |
|  | 2006-2010 | **1.59 (1.24, 2.04)** | **0.35 (0.24, 0.53)** | 0.91 (0.74, 1.13) | 0.83 (0.67, 1.03) | **0.76 (0.63, 0.93)** | 1.11 (0.90, 1.37) |
|  | 2011-2015 | **1.81 (1.42, 2.32)** | **0.61 (0.40, 0.92)** | 0.91 (0.74, 1.13) | 0.89 (0.72, 1.10) | 0.91 (0.75, 1.09) | 0.92 (0.75, 1.13) |
|  | 2016-2021 | **1.38 (1.10, 1.75)** | 0.85 (0.57, 1.27) | **1.44 (1.18, 1.77)** | **0.66 (0.54, 0.81)** | 1.05 (0.87, 1.25) | 0.96 (0.79, 1.17) |
| Age Group (years; vs. 40-64) | | | | | | | |
|  | 20-39 | 1.23 (0.95, 1.58) | 0.87 (0.59, 1.29) | **0.47 (0.38, 0.58)** | **3.17 (2.51, 4.01)** | 0.97 (0.80, 1.17) | **1.86 (1.51, 2.29)** |
|  | 65+ | **1.19 (1.04, 1.37)** | **0.70 (0.56, 0.87)** | **1.13 (1.01, 1.26)** | **0.27 (0.24, 0.30)** | 0.65 (0.59, 0.72) | **0.69 (0.62, 0.77)** |
| Marital status at diagnosis (vs. Married/Partnered) | | | | | | | |
|  | Not Partnered | **0.74 (0.65, 0.84)** | 0.80 (0.63, 1.01) | 1.05 (0.94, 1.17) | **0.79 (0.71, 0.89)** | **0.87 (0.78, 0.96)** | **1.15 (1.03, 1.29)** |
|  | Unknown | **0.59 (0.47, 0.74)** | **0.37 (0.27, 0.50)** | 1.13 (0.94, 1.35) | **0.55 (0.46, 0.66)** | **0.71 (0.60, 0.83)** | 0.94 (0.78, 1.13) |
| PRCDA (vs. PRCDA county) | | | | | | | |
|  | No | **1.50 (1.27, 1.76)** | 1.00 (0.74, 1.35) | 1.05 (0.90, 1.21) | 1.08 (0.93, 1.26) | 0.89 (0.78, 1.02) | 0.91 (0.79, 1.06) |
| Ethnicity (vs. Hispanic) | | | | | | | |
|  | Non-Hispanic | 1.07 (0.85, 1.33) | 1.23 (0.82, 1.84) | **1.24 (1.01, 1.52)** | 0.86 (0.70, 1.06) | 1.06 (0.88, 1.28) | 0.90 (0.73, 1.09) |
| Stage (vs. Local) | | | | | | | |
|  | Regional | 0.98 (0.85, 1.14) | **1.64 (1.16, 2.32)** | **0.39 (0.35, 0.44)** | **5.53 (4.87, 6.28)** | **1.41 (1.26, 1.57)** |  |
|  | Distant | 1.16 (0.85, 1.60) | **0.33 (0.23, 0.47)** | **0.30 (0.21, 0.44)** | **3.67 (2.93, 4.60)** | **0.54 (0.43, 0.67)** |  |
|  | Unknown/Unstaged | 0.95 (0.74, 1.24) | **0.08 (0.06, 0.12)** | **0.67 (0.53, 0.84)** | **1.33 (1.07, 1.65)** | **0.75 (0.62, 0.91)** |  |

^a^ Statistically significant associations given in **bold**.

^b^Patients underwent at least one documented treatment, including chemotherapy, radiation, or cancer-directed surgery. A total of 6,775 individuals were included in this analysis.

^c^Analysis included only patients who underwent cancer-directed surgery. A total of 6,493 individuals were included in this analysis. The reference category here was mastectomy with or without reconstruction/bi-lateral mastectomy with or without reconstruction.

**Supplementary Table 6**. Full results of multivariable-adjusted models showing associations of region with components of treatment received for breast cancer among AIAN women, including region and all covariates, among the subset of women with breast cancer subtype information. Data from the Surveillance, Epidemiology, and End Results program, 2010-2021, n = 4,647.^a^

| **Characteristic** | **Time to Treatment ≤60 days**^b^ | **ER/PR testing occurred** | **Lumpectomy, or other breast preserving surgery**^c^ | **Chemotherapy** | **Radiation** | **Diagnosed at late stage**^d^ |
| --- | --- | --- | --- | --- | --- | --- |
| PRCDA Region (vs. Pacific Coast) | | | | | | |
| Alaska | **3.93 (2.90, 5.32)** | 1.54 (0.84, 2.81) | **0.82 (0.68, 0.99)** | **1.28 (1.04, 1.59)** | 1.04 (0.87, 1.24) | 0.92 (0.77, 1.10) |
| East | **1.79 (1.29, 2.50)** | 1.43 (0.55, 3.76) | **0.71 (0.54, 0.92)** | 0.97 (0.72, 1.31) | 0.86 (0.68, 1.09) | 1.06 (0.83, 1.35) |
| Northern Plains | **4.55 (1.64, 12.64)** | 1.90 (0.20, 17.75) | 0.91 (0.53, 1.55) | 1.09 (0.58, 2.04) | 1.24 (0.74, 2.06) | 0.73 (0.42, 1.25) |
| Southwest | **1.33 (1.08, 1.64)** | 0.40 (0.27, 0.60) | 1.08 (0.90, 1.30) | 0.96 (0.78, 1.17) | **0.81 (0.69, 0.95)** | 1.00 (0.85, 1.18) |
| Diagnosis Year (vs. 2010) | | | | | | |
| 2011-2015 | 0.96 (0.69, 1.34) | 1.04 (0.55, 1.94) | 1.24 (0.95, 1.62) | 0.92 (0.68, 1.24) | 1.16 (0.91, 1.49) | **0.73 (0.57, 0.94)** |
| 2016-2021 | 0.74 (0.53, 1.03) | 1.54 (0.83, 2.87) | **1.96 (1.51, 2.55)** | **0.66 (0.49, 0.89)** | **1.31 (1.03, 1.67)** | **0.78 (0.61, 0.99)** |
| Age Group (vs. 40-64) | | | | | | |
| 20-39 | 1.21 (0.86, 1.69) | 1.27 (0.59, 2.73) | **0.43 (0.33, 0.57)** | **2.32 (1.65, 3.25)** | 0.96 (0.74, 1.23) | **1.89 (1.47, 2.42)** |
| 65+ | 1.17 (0.99, 1.39) | 0.90 (0.62, 1.30) | **1.27 (1.10, 1.47)** | **0.28 (0.24, 0.33)** | **0.68 (0.60, 0.77)** | **0.67 (0.59, 0.77)** |
| Ethnicity (vs. Hispanic) | | | | | | |
| Non-Hispanic | 1.27 (0.98, 1.64) | 1.00 (0.50, 2.00) | 1.23 (0.96, 1.57) | **0.73 (0.56, 0.95)** | 1.00 (0.80, 1.25) | 0.87 (0.70, 1.09) |
| Marital status at diagnosis (vs. Married/Partnered) | | | | | | |
| Not Partnered | **0.71 (0.60, 0.84)** | 0.83 (0.55, 1.24) | 1.04 (0.90, 1.19) | 0.86 (0.73, 1.00) | **0.82 (0.73, 0.94)** | **1.21 (1.07, 1.38)** |
| Unknown | **0.61 (0.46, 0.81)** | **0.40 (0.24, 0.64)** | 1.17 (0.93, 1.49) | **0.57 (0.44, 0.75)** | **0.79 (0.64, 0.97)** | 0.98 (0.79, 1.21) |
| Non-PRCDA vs PRCDA | 1.03 (0.84, 1.27) | 1.60 (0.89, 2.86) | 1.03 (0.86, 1.24) | **1.24 (1.01, 1.52)** | 0.95 (0.80, 1.12) | 0.95 (0.80, 1.13) |
| Breast Subtype (vs. HR+/HER2+) | | | | | | |
| HR+/HER2- | 0.85 (0.66, 1.11) |  | **1.58 (1.28, 1.95)** | **0.10 (0.07, 0.12)** | **1.58 (1.30, 1.92)** | **0.69 (0.57, 0.84)** |
| HR-/HER2+ | 0.96 (0.62, 1.50) |  | 0.78 (0.54, 1.12) | 0.98 (0.63, 1.51) | 1.08 (0.78, 1.49) | 1.28 (0.93, 1.76) |
| HR-/HER2- | 1.03 (0.73, 1.47) |  | 1.17 (0.88, 1.55) | 1.14 (0.81, 1.61) | 1.10 (0.85, 1.43) | 0.83 (0.64, 1.08) |
| No/Unknown | 0.72 (0.48, 1.10) |  | **2.07 (1.42, 3.02)** | **0.07 (0.05, 0.10)** | **0.62 (0.45, 0.86)** | 0.86 (0.63, 1.18) |
| Stage (vs. Local) | | | | | | |
| Regional | 0.94 (0.79, 1.11) | 1.44 (0.88, 2.36) | **0.40 (0.35, 0.46)** | **6.68 (5.67, 7.88)** | **1.60 (1.40, 1.83)** |  |
| Distant | 1.34 (0.91, 1.98) | **0.20 (0.13, 0.31)** | **0.22 (0.13, 0.38)** | **4.24 (3.17, 5.68)** | **0.58 (0.45, 0.75)** |  |
| Unknown/Unstaged | **0.37 (0.15, 0.90)** | **0.01 (0.01, 0.03)** | 0.42 (0.16, 1.12) | **0.42 (0.18, 0.98)** | **0.04 (0.01, 0.17)** |  |

^a^Statistically significant associations given in **bold**.

^b^Patients received at least one documented treatment, including chemotherapy, radiation, or cancer-directed surgery. A total of 4,315 individuals were included in these analyses.

^c^Analysis included only patients that underwent cancer-directed surgery. A total of 4,098 individuals were included in the study. Reference is Mastectomy with or without reconstruction/Bi-lateral Mastectomy with or without reconstruction.

^d^Late stage defined as stage at regional or distant.

**Supplementary Table 7**. Frequencies of individuals receiving components of care among AIAN people with lung cancer, by region, 2000-2021. Data from the Surveillance, Epidemiology, and End Results program, 2000-2021, n = 5,403.

| **Category** | **Alaska (N=1423)** | **East (N=449)** | **Northern Plains (N=107)** | **Pacific Coast (N=2957)** | **Southwest (N=467)** |
| --- | --- | --- | --- | --- | --- |
| Time to Treatment [IQR]^a^ | | | | | |
|  | 30 [14, 56] | 23 [6, 46] | 21 [9, 32.5] | 31 [11, 58] | 29 [11, 56] |
| Time to Treatment ≤60 days^a^ | | | | | |
| Yes | 731 (77.6%) | 245 (80.1%) | 55 (73.3%) | 1250 (63.3%) | 197 (74.6%) |
| No | 200 (21.2%) | 50 (16.3%) | 5 (6.7%) | 390 (19.7%) | 57 (21.6%) |
| Unknown | 11 (1.2%) | 11 (3.6%) | 15 (20%) | 335 (17%) | 10 (3.8%) |
| Microscopic Confirmation (%) | | | | | |
| Yes | 1248 (87.7%) | 403 (89.8%) | 95 (88.8%) | 2612 (88.3%) | 419 (89.7%) |
| No/Unknown | 175 (12.3%) | 46 (10.2%) | 12 (11.2%) | 345 (11.7%) | 48 (10.3%) |
| Chemotherapy (%)^b^ | | | | | |
| Yes | 584 (41%) | 188 (41.9%) | 51 (47.7%) | 1160 (39.2%) | 175 (37.5%) |
| No/Unknown | 839 (59%) | 261 (58.1%) | 56 (52.3%) | 1797 (60.8%) | 292 (62.5%) |
| Radiation (%)^b^ | | | | | |
| Yes | 652 (45.8%) | 167 (37.2%) | 38 (35.5%) | 1103 (37.3%) | 129 (27.6%) |
| No/Unknown | 771 (54.2%) | 282 (62.8%) | 69 (64.5%) | 1854 (62.7%) | 338 (72.4%) |
| Cancer-directed Surgery (%) | | | | | |
| Surgery performed | 187 (13.1%) | 94 (20.9%) | 22 (20.6%) | 559 (18.9%) | 66 (14.1%) |
| Not recommended | 1077 (75.7%) | 327 (72.8%) | 82 (76.6%) | 2266 (76.6%) | 278 (59.5%) |
| Recommended but not performed | 149 (10.5%) | 18 (4%) | <5 | 106 (3.6%) | 107 (22.9%) |
| Unknown | 10 (0.7%) | 10 (2.2%) | 0 (0%) | 26 (0.9%) | 16 (3.4%) |
| Receipt of Surgery (%) | | | | | |
| Surgery received | 123 (11.6%) | 79 (22.2%) | 17 (19.8%) | 425 (18.7%) | 44 (12.4%) |
| No/Unknown | 936 (88.4%) | 277 (77.8%) | 69 (80.2%) | 1851 (81.3%) | 310 (87.6%) |
| Diagnosed at late stage (%)^c^ | | | | | |
| Yes | 1010 (80.6%) | 304 (73.8%) | 85 (88.5%) | 1976 (75%) | 290 (69.7%) |
| No | 197 (15.7%) | 88 (21.4%) | 9 (9.4%) | 521 (19.8%) | 77 (18.5%) |
| Unknown | 46 (3.7%) | 20 (4.9%) | <5 | 137 (5.2%) | 49 (11.8%) |

^a^Patients received at least one documented treatment, including chemotherapy, radiation, or cancer-directed surgery. A total of 3,562 individuals were included in these analyses.

^b^Due to known issues of sensitivity in the chemotherapy and radiation variables (Noone et al. 2016), it is not recommended to interpret these data as reflecting prevalence of treatment receipt.

^c^SEER Combined Stage available from 2004 onwards. A total of 4,811 individuals were included in this analysis. Late stage was defined as regional or distant stage.

**Supplementary Table 8**. Full results of multivariable-adjusted models showing associations of region with components of treatment received for lung cancer among AIAN people, including region and all covariates; Surveillance, Epidemiology and Results program, 2000-2021, n = 5,403.^a^

| **Characteristic** | **Level** | **Time to Treatment within 60 days^b^** | **Microscopic Confirmation** | **Receipt of Surgery** | **Chemotherapy** | **Radiation** | **Diagnosed at late stage^c^** |
| --- | --- | --- | --- | --- | --- | --- | --- |
| PRCDA Region (vs. Pacific Coast) | | | | | | | |
|  | Alaska | **2.25 (1.85, 2.74)** | 0.87 (0.70, 1.09) | **0.70 (0.56, 0.87)** | 1.01 (0.87, 1.17) | **1.43 (1.24, 1.64)** | **1.32 (1.08, 1.62)** |
|  | East | **1.86 (1.34, 2.59)** | 1.04 (0.72, 1.52) | 0.87 (0.63, 1.19) | 1.26 (0.98, 1.61) | 1.16 (0.92, 1.47) | 0.95 (0.71, 1.28) |
|  | Northern Plains | 1.19 (0.69, 2.04) | 0.95 (0.50, 1.81) | 1.18 (0.65, 2.12) | 1.33 (0.88, 2.02) | 1.00 (0.66, 1.52) | **2.44 (1.21, 4.94)** |
|  | Southwest | **1.75 (1.29, 2.36)** | 1.37 (0.97, 1.93) | 0.76 (0.55, 1.05) | 1.02 (0.82, 1.28) | **0.66 (0.53, 0.82)** | 0.94 (0.71, 1.24) |
| Diagnosis Year (vs. 2001-2005) | | | | | | | |
|  | 2006-2010 | 1.13 (0.85, 1.49) | **0.29 (0.21, 0.40)** | **0.67 (0.50, 0.90)** | **0.58 (0.47, 0.72)** | **0.65 (0.53, 0.80)** | 0.94 (0.69, 1.29) |
|  | 2011-2015 | **1.98 (1.49, 2.64)** | **0.31 (0.23, 0.43)** | **0.59 (0.44, 0.79)** | **0.64 (0.52, 0.79)** | 0.82 (0.67, 1.00) | 0.83 (0.61, 1.13) |
|  | 2016-2021 | **1.43 (1.09, 1.87)** | **0.37 (0.27, 0.51)** | **0.45 (0.34, 0.60)** | **0.56 (0.46, 0.69)** | 0.83 (0.69, 1.01) | **0.64 (0.48, 0.86)** |
| Age Group (years; vs. 40-65) | | | | | | | |
|  | 20-39 | 1.51 (0.59, 3.87) | 2.85 (0.38, 21.45) | **2.55 (1.15, 5.62)** | 0.68 (0.35, 1.31) | 1.27 (0.66, 2.41) | 3.23 (0.76, 13.73) |
|  | 65+ | **0.77 (0.66, 0.89)** | **0.38 (0.31, 0.47)** | **0.46 (0.39, 0.55)** | **0.46 (0.40, 0.52)** | **0.72 (0.64, 0.81)** | 0.87 (0.74, 1.02) |
| Marital status at diagnosis (vs. married) | | | | | | | |
|  | Not Partnered | 0.87 (0.74, 1.02) | **0.57 (0.46, 0.69)** | **0.66 (0.55, 0.79)** | **0.64 (0.57, 0.73)** | 0.90 (0.80, 1.01) | 1.18 (1.00, 1.39) |
|  | Unknown | 0.89 (0.66, 1.20) | 0.73 (0.53, 1.00) | 0.66 (0.48, 0.91) | **0.54 (0.44, 0.67)** | **0.61 (0.50, 0.75)** | 1.22 (0.91, 1.64) |
| PRCDA (vs. PRCDA counties) | | | | | | | |
|  | No | **1.52 (1.22, 1.91)** | 1.03 (0.79, 1.34) | **1.44 (1.14, 1.81)** | **0.80 (0.67, 0.96)** | **0.73 (0.61, 0.86)** | 0.93 (0.74, 1.16) |
| Sex (vs. male) | | | | | | | |
|  | Female | 1.01 (0.86, 1.17) | 1.04 (0.87, 1.24) | **1.19 (1.01, 1.40)** | 1.05 (0.94, 1.19) | 0.97 (0.87, 1.09) | **0.71 (0.61, 0.83)** |
| Ethnicity (vs. Hispanic) | | | | | | | |
|  | Non-Hispanic | 0.75 (0.49, 1.13) | 0.83 (0.51, 1.34) | 0.92 (0.61, 1.39) | 1.12 (0.82, 1.54) | 1.06 (0.78, 1.43) | 1.29 (0.90, 1.84) |
| Stage at diagnosis (vs. Local) | | | | | | | |
|  | Distant | **3.00 (2.45, 3.67)** | 0.77 (0.58, 1.01) | **0.03 (0.02, 0.04)** | **7.53 (5.94, 9.53)** | **1.18 (1.00, 1.40)** |  |
|  | Regional | **1.80 (1.44, 2.24)** | 1.39 (0.97, 1.99) | **0.43 (0.35, 0.53)** | **11.28 (8.73, 14.57)** | **1.89 (1.56, 2.29)** |  |
|  | Unknown/Unstaged | **1.65 (1.19, 2.29)** | **0.14 (0.10, 0.20)** | **0.11 (0.08, 0.14)** | **2.43 (1.80, 3.27)** | **0.66 (0.52, 0.84)** |  |

^a^No event was the reference group for all outcomes. The model was adjusted for covariates, including Diagnosis Year, Age Group, Sex, Ethnicity, Marital Status at Diagnosis, stage (except detected at late stage), and PRCDA. Statistically significant associations given in **bold**.

^b^Patients received at least one documented treatment, including chemotherapy, radiation, or cancer-directed surgery. A total of 3,671 individuals were included in these analyses.

^c^SEER derived summary stage available from 2004 onwards. A total of 4,811 individuals were included in this analysis.  Late stage diagnosis is defined as diagnosis at regional or distant stage.

**Supplementary Table 9**. Frequencies of individuals receiving components of care among AIAN people with colorectal cancer, by region, 2000-2021. Data from the Surveillance, Epidemiology, and End Results program, n = 5,626.

| **Category** | **Alaska (N=1472)** | **East (N=355)** | **Northern Plains (N=80)** | **Pacific Coast (N=2379)** | **Southwest (N=1340)** |
| --- | --- | --- | --- | --- | --- |
| Time to Treatment [IQR]^a^ | | | | | |
|  | 11 [0, 26.5] | 8 [0, 30] | 12 [2, 33] | 15 [0, 36] | 14 [0, 36] |
| Time to Treatment ≤60 days (%)^a^ | | | | | |
| Yes | 1280 (95%) | 287 (92.3%) | 49 (69%) | 1663 (77.7%) | 998 (87.9%) |
| No | 56 (4.2%) | 19 (6.1%) | 6 (8.5%) | 194 (9.1%) | 126 (11.1%) |
| Unknown | 12 (0.9%) | 5 (1.6%) | 16 (22.5%) | 283 (13.2%) | 12 (1.1%) |
| Microscopic Confirmation (%) | | | | | |
| Yes | 1436 (97.6%) | 342 (96.3%) | 79 (98.8%) | 2323 (97.6%) | 1305 (97.4%) |
| No/Unknown | 36 (2.4%) | 13 (3.7%) | <5 | 56 (2.4%) | 35 (2.6%) |
| Chemotherapy (%)^b^ | | | | | |
| Yes | 575 (39.1%) | 138 (38.9%) | 36 (45%) | 996 (41.9%) | 577 (43.1%) |
| No/Unknown | 897 (60.9%) | 217 (61.1%) | 44 (55%) | 1383 (58.1%) | 763 (56.9%) |
| Radiation (%)^b^ | | | | | |
| Yes | 271 (18.4%) | 53 (14.9%) | 18 (22.5%) | 396 (16.6%) | 237 (17.7%) |
| No/Unknown | 1201 (81.6%) | 302 (85.1%) | 62 (77.5%) | 1983 (83.4%) | 1103 (82.3%) |
| Cancer-directed Surgery (%) | | | | | |
| Surgery performed | 1227 (83.4%) | 280 (78.9%) | 65 (81.3%) | 1902 (79.9%) | 1003 (74.9%) |
| Not recommended | 203 (13.8%) | 65 (18.3%) | 11 (13.8%) | 409 (17.2%) | 208 (15.5%) |
| Recommended but not performed | 40 (2.7%) | 10 (2.8%) | <5 | 65 (2.7%) | 114 (8.5%) |
| Unknown | <5 | 0 (0%) | 0 (0%) | <5 | 15 (1.1%) |
| Receipt of Surgery-Colon (%)^c^ | | | | | |
| Yes | 876 (86.3%) | 178 (79.5%) | 39 (86.7%) | 1265 (83.2%) | 664 (77.8%) |
| No/Unknown | 139 (13.7%) | 46 (20.5%) | 6 (13.3%) | 255 (16.8%) | 189 (22.2%) |
| Type of Surgery-Colon(%)^c^ | | | | | |
| Minor surgery | 47 (4.6%) | 7 (3.1%) | <5 | 62 (4.1%) | 35 (4.1%) |
| Partial colectomy | 321 (31.6%) | 64 (28.6%) | 19 (42.2%) | 515 (33.9%) | 258 (30.2%) |
| Subtotal or total colectomy | 508 (50%) | 107 (47.8%) | 19 (42.2%) | 688 (45.3%) | 371 (43.5%) |
| No/Unknown | 139 (13.7%) | 46 (20.5%) | 6 (13.3%) | 255 (16.8%) | 189 (22.2%) |
| Receipt of Surgery-Rectum (%)^c^ | | | | | |
| Yes | 337 (75.9%) | 81 (72.3%) | 26 (74.3%) | 580 (71.8%) | 305 (66.3%) |
| No/Unknown | 107 (24.1%) | 31 (27.7%) | 9 (25.7%) | 228 (28.2%) | 155 (33.7%) |
| Type of Surgery-Rectum(%)^c^ | | | | | |
| Minor surgery | 97 (21.8%) | 15 (13.4%) | <5 | 128 (15.8%) | 69 (15%) |
| Wedge or segmental resection; pull through with sphincter preservation | 188 (42.3%) | 59 (52.7%) | 17 (48.6%) | 356 (44.1%) | 179 (38.9%) |
| Total proctectomy or proctolectomy | 52 (11.7%) | 7 (6.3%) | 6 (17.1%) | 96 (11.9%) | 57 (12.4%) |
| No/Unknown | 107 (24.1%) | 31 (27.7%) | 9 (25.7%) | 228 (28.2%) | 155 (33.7%) |
| Regional Nodes 12+ examined (%) | | | | | |
| Yes | 644 (43.8%) | 188 (53%) | 38 (47.5%) | 1257 (52.8%) | 595 (44.4%) |
| No/Unknown | 828 (56.3%) | 167 (47%) | 42 (52.5%) | 1122 (47.2%) | 745 (55.6%) |
| KRAS testing results (%)^d^ | | | | | |
| Abnormal | 18 (24.7%) | <5 | 0 (0%) | 16 (12.2%) | 23 (29.5%) |
| Missing or Invalid | <5 | 0 (0%) | 0 (0%) | <5 | <5 |
| Normal (wild type) | 23 (31.5%) | 6 (26.1%) | <5 | 30 (22.9%) | 16 (20.5%) |
| Unknown | 30 (41.1%) | 15 (65.2%) | <5 | 81 (61.8%) | 38 (48.7%) |
| KRAS testing documented (%)^d^ | | | | | |
| Yes | 41 (56.2%) | 8 (34.8%) | <5 | 46 (35.1%) | 39 (50%) |
| No/Unknown | 32 (43.8%) | 15 (65.2%) | <5 | 85 (64.9%) | 39 (50%) |
| MSI testing(%)^d^ | | | | | |
| MSI Stable | 128 (40.9%) | 46 (46.5%) | 10 (47.6%) | 300 (50.4%) | 150 (43.2%) |
| MSI Unstable | 30 (9.6%) | 5 (5.1%) | <5 | 44 (7.4%) | 13 (3.7%) |
| Missing or Invalid | 17 (5.4%) | 12 (12.1%) | 0 (0%) | 40 (6.7%) | 19 (5.5%) |
| Unknown | 138 (44.1%) | 36 (36.4%) | 8 (38.1%) | 211 (35.5%) | 165 (47.6%) |
| MSI testing documented (%)^d^ | | | | | |
| Yes | 158 (50.5%) | 51 (51.5%) | 13 (61.9%) | 344 (57.8%) | 163 (47%) |
| No/Unknown | 155 (49.5%) | 48 (48.5%) | 8 (38.1%) | 251 (42.2%) | 184 (53%) |
| Diagnosed at late stage (%)^e^ | | | | | |
| Yes | 788 (59.7%) | 208 (61.2%) | 38 (52.8%) | 1290 (58.9%) | 725 (58%) |
| No | 501 (38%) | 114 (33.5%) | 30 (41.7%) | 789 (36%) | 431 (34.5%) |
| Unknown | 31 (2.3%) | 18 (5.3%) | <5 | 112 (5.1%) | 95 (7.6%) |

^a^Patients received at least one documented treatment, including chemotherapy, radiation, or cancer-directed surgery. A total of 4,915 individuals were included in this analysis.

^b^Due to known issues of sensitivity in the chemotherapy and radiation variables (Noone et al. 2016), it is not recommended to interpret these data as reflecting prevalence of treatment receipt.

^c^A total of 3,657 participants were included in analysis of receipt of surgery for colon cancer, and 1,859 individuals were included in analysis of receipt of surgery for rectal cancer.

^d^KRAS and MSI information available since 2018. KRAS testing information collected for stage IV patients only. A total of 309 individuals were included in analysis of KRAS and 1,375 individuals were included in the MSI analysis.

^e^SEER Combined Stage available from 2004 onwards. A total of 5,174 individuals were included in this analysis. Late stage was defined as diagnosis at regional or distant stage

**Supplementary Table 10**. Full results of multivariable-adjusted models showing associations of region with components of treatment received for colorectal cancer among AIAN people, including region and all covariates; Surveillance, Epidemiology and Results program, 2000-2021, n = 5,626.^a^

| **Characteristic** | **Level** | **Time to Treatment ≤60 days^b^** | **Microscopic Confirmation** | **12 Regional Nodes examined** | **MSI testing documented^c^** | **Receipt of Surgery, Colon only**^d^ | **Receipt of Surgery, Rectum only^d^** | **Chemotherapy** | **Radiation** | **Diagnosed at late stage^e^** |
| --- | --- | --- | --- | --- | --- | --- | --- | --- | --- | --- |
| PRCDA Region (vs. Pacific Coast) | | | | | | | | | | |
|  | Alaska | **5.49 (4.14, 7.27)** | 0.86 (0.53, 1.38) | **0.70 (0.60, 0.82)** | **0.69 (0.51, 0.95)** | 1.17 (0.86, 1.58) | 1.30 (0.93, 1.83) | 0.99 (0.84, 1.18) | **1.34 (1.10, 1.62)** | 1.03 (0.88, 1.20) |
|  | East | **3.11 (1.98, 4.88)** | 0.58 (0.28, 1.20) | 0.95 (0.73, 1.24) | 0.72 (0.44, 1.17) | 1.00 (0.63, 1.60) | 1.25 (0.72, 2.17) | 0.80 (0.60, 1.07) | 0.80 (0.56, 1.13) | 1.22 (0.93, 1.59) |
|  | Northern Plains | 0.63 (0.37, 1.06) | 1.62 (0.21, 12.74) | 0.76 (0.47, 1.23) | 1.74 (0.66, 4.60) | 1.52 (0.52, 4.49) | 1.12 (0.46, 2.75) | 1.38 (0.81, 2.36) | 1.52 (0.87, 2.67) | 0.77 (0.47, 1.27) |
|  | Southwest | **2.01 (1.62, 2.49)** | 1.00 (0.62, 1.60) | **0.68 (0.59, 0.79)** | **0.64 (0.48, 0.85)** | **0.65 (0.49, 0.86)** | **0.72 (0.54, 0.98)** | 1.01 (0.86, 1.19) | 1.09 (0.90, 1.32) | 1.04 (0.89, 1.21) |
| Diagnosis Year (vs. 2001-2005) | | | | | | | | | | |
|  | 2006-2010 | **1.44 (1.06, 1.95)** | **0.31 (0.16, 0.61)** | 1.02 (0.82, 1.26) |  | **0.16 (0.11, 0.25)** | **0.28 (0.17, 0.46)** | **0.76 (0.60, 0.97)** | 1.06 (0.81, 1.40) | 1.10 (0.87, 1.38) |
|  | 2011-2015 | **2.32 (1.70, 3.16)** | **0.19 (0.10, 0.34)** | 1.22 (0.99, 1.50) |  | **0.11 (0.07, 0.17)** | **0.25 (0.15, 0.40)** | 0.82 (0.65, 1.03) | 1.06 (0.81, 1.38) | 1.03 (0.83, 1.29) |
|  | 2016-2021 | **1.71 (1.28, 2.28)** | **0.20 (0.11, 0.35)** | **1.55 (1.27, 1.89)** |  | **0.08 (0.06, 0.13)** | **0.18 (0.12, 0.29)** | **0.76 (0.61, 0.95)** | 0.94 (0.72, 1.21) | 1.21 (0.97, 1.50) |
| Age Group (years; vs. 40-64) | | | | | | | | | | |
|  | 20-39 | **1.80 (1.17, 2.75)** | 3.93 (0.53, 29.26) | 1.09 (0.84, 1.41) | 1.32 (0.77, 2.24) | 1.55 (0.89, 2.71) | **0.53 (0.33, 0.86)** | **1.48 (1.11, 1.96)** | 0.98 (0.73, 1.32) | 1.19 (0.90, 1.56) |
|  | 65+ | **1.18 (1.00, 1.40)** | **0.28 (0.18, 0.42)** | 0.94 (0.84, 1.06) | **0.79 (0.63, 0.99)** | **0.63 (0.50, 0.78)** | **0.69 (0.54, 0.88)** | **0.36 (0.32, 0.41)** | **0.48 (0.41, 0.56)** | 0.99 (0.88, 1.12) |
| Marital status at diagnosis (vs. Married/Partnered) | | | | | | | | | | |
|  | Not Partnered | **0.80 (0.67, 0.96)** | **0.47 (0.30, 0.72)** | **0.80 (0.71, 0.91)** | **0.78 (0.61, 0.99)** | **0.51 (0.40, 0.66)** | **0.65 (0.50, 0.84)** | **0.77 (0.67, 0.88)** | 0.93 (0.80, 1.09) | **1.29 (1.14, 1.47)** |
|  | Unknown | 0.98 (0.73, 1.32) | 0.60 (0.34, 1.05) | **0.70 (0.59, 0.84)** | 0.85 (0.60, 1.19) | **0.49 (0.36, 0.69)** | **0.65 (0.45, 0.93)** | **0.47 (0.39, 0.58)** | **0.60 (0.47, 0.76)** | 0.84 (0.70, 1.01) |
| PRCDA (vs. PRCDA counties) | | | | | | | | | | |
|  | No | 1.06 (0.84, 1.34) | 1.31 (0.72, 2.38) | 1.01 (0.84, 1.21) | 1.03 (0.72, 1.46) | 0.71 (0.51, 1.00) | 0.94 (0.65, 1.36) | 0.99 (0.81, 1.20) | 1.14 (0.91, 1.44) | 0.89 (0.74, 1.07) |
| Sex (vs. Male) | | | | | | | | | | |
|  | Female | 1.16 (0.99, 1.37) | 1.02 (0.72, 1.46) | 1.09 (0.97, 1.22) | 1.01 (0.81, 1.26) | 1.19 (0.96, 1.48) | 0.88 (0.70, 1.11) | 0.79 (0.70, 0.89) | **0.68 (0.59, 0.79)** |  |
| Ethnicity | | | | | | | | | | |
|  | Non-Hispanic | 1.12 (0.84, 1.50) | 0.95 (0.44, 2.05) | 1.05 (0.84, 1.32) | 0.89 (0.60, 1.32) | 1.32 (0.85, 2.03) | 0.84 (0.54, 1.32) | 1.04 (0.81, 1.33) | 1.04 (0.78, 1.39) | 0.99 (0.79, 1.25) |
| Stage (vs. Local)^e^ | | | | | | | | | | |
|  | Distant | 0.94 (0.74, 1.19) | **0.14 (0.07, 0.26)** | **0.50 (0.42, 0.58)** | **1.44 (1.07, 1.92)** | **0.05 (0.04, 0.07)** | **0.07 (0.05, 0.10)** | **12.83 (10.60, 15.53)** | **1.67 (1.34, 2.09)** |  |
|  | Regional | 1.16 (0.95, 1.43) | 2.14 (0.80, 5.72) | **2.38 (2.07, 2.73)** | **1.59 (1.22, 2.06)** | **2.67 (1.67, 4.28)** | **0.71 (0.51, 0.97)** | **12.02 (10.09, 14.33)** | **3.32 (2.74, 4.01)** |  |
|  | Unknown/Unstaged | 0.92 (0.67, 1.27) | **0.04 (0.02, 0.08)** | **0.36 (0.29, 0.45)** | **0.19 (0.10, 0.37)** | **0.04 (0.02, 0.06)** | **0.13 (0.08, 0.20)** | **2.70 (2.11, 3.46)** | **1.64 (1.23, 2.20)** |  |

^a^No event was the reference group for all outcomes. The model was adjusted for covariates, including Diagnosis Year, Age Group, Sex, Ethnicity, Marital Status at Diagnosis, stage (except detected at late stage), and PRCDA.

^b^Patients underwent at least one documented treatment, including chemotherapy, radiation, or cancer-directed surgery. A total of 4,915 participants were included in these analyses.

^c^MSI testing from 2018 onwards. A total of 1,393 participants were included in MSI analyses.

**^d^**A total of 3,657 participants were included in analyses of Receipt of Surgery-Colon, and a total of 1,859 participants were included in analyses of Receipt of Surgery-Rectum

^e^SEER derived summary stage from 2004 onwards. A total of 5,174 participants were included in these analyses. Late stage diagnosis is defined as the stage at the regional or distant stage.

**Supplementary Table 11**. Unadjusted and multivariable-adjusted associations of region with components of treatment received for colon cancer only, among AIAN people; Surveillance, Epidemiology and Results program, 2000-2021, n = 3,657.

| **Region^a^** | **Treatment Time ≤60 days^b^** | **Microscopic Confirmation** | **12 Regional Nodes Examined** | **MSI testing documented^c^** | **Chemotherapy** | **Radiation** | **Diagnosed at late stage^d^** |
| --- | --- | --- | --- | --- | --- | --- | --- |
| Unadjusted | | | | | | | |
| Alaska | **7.09 (4.84, 10.40)** | 1.05 (0.66, 1.67) | **0.66 (0.56, 0.78)** | 0.81 (0.57, 1.14) | 1.00 (0.85, 1.18) | **1.78 (1.08, 2.94)** | 0.98 (0.82, 1.17) |
| East | **5.61 (2.73, 11.54)** | 0.62 (0.32, 1.21) | 0.93 (0.70, 1.24) | 1.02 (0.58, 1.79) | 0.93 (0.69, 1.25) | 1.66 (0.72, 3.82) | 1.09 (0.80, 1.49) |
| Northern Plains | 0.55 (0.28, 1.10) | 1.41 (0.19, 10.43) | 0.75 (0.42, 1.37) | 1.78 (0.47, 6.83) | 1.08 (0.58, 2.01) | 1.17 (0.16, 8.75) | 0.67 (0.35, 1.30) |
| Southwest | **2.18 (1.65, 2.87)** | 0.88 (0.55, 1.40) | **0.65 (0.55, 0.77)** | 0.74 (0.53, 1.04) | 1.07 (0.90, 1.28) | 1.04 (0.57, 1.91) | 1.05 (0.87, 1.27) |
| Adjusted^e^ | | | | | | | |
| Alaska | 7.05 (4.71, 10.55) | 0.93 (0.55, 1.58) | **0.65 (0.53, 0.79)** | 0.81 (0.54, 1.21) | 1.13 (0.90, 1.40) | **1.76 (1.00, 3.08)** | 0.99 (0.82, 1.21) |
| East | 5.17 (2.45, 10.90) | 0.58 (0.26, 1.28) | 0.93 (0.66, 1.31) | 0.86 (0.45, 1.68) | 0.89 (0.61, 1.31) | 1.48 (0.57, 3.86) | 1.29 (0.91, 1.82) |
| Northern Plains | 0.51 (0.25, 1.06) | 1.22 (0.14, 10.33) | 0.71 (0.37, 1.37) | 3.24 (0.68, 15.33) | 1.40 (0.66, 2.95) | 1.50 (0.19, 11.87) | 0.71 (0.36, 1.40) |
| Southwest | **2.04 (1.53, 2.73)** | 1.03 (0.61, 1.73) | **0.57 (0.47, 0.70)** | 0.79 (0.55, 1.15) | 1.02 (0.82, 1.26) | 1.04 (0.55, 1.97) | 1.01 (0.83, 1.23) |

^a^Pacific Coast was the region of reference for all analyses. No event was the reference group for all outcomes. Statistically significant associations are given in **bold**.

^b^Patients underwent at least one documented treatment, including chemotherapy, radiation, or cancer-directed surgery. A total of 1,311 participants were included.

^c^MSI testing from 2018 onwards. A total of 865 participants were included in MSI analyses.

^d^SEER derived summary stage from 2004 onwards. A total of 3,337 participants were included.

^e^The model was adjusted for covariates including Diagnosis Year, Age Group, Sex, Ethnicity, Marital Status at Diagnosis, stage (except diagnosed at late stage), and PRCDA

**Supplementary Table 12**. Unadjusted and multivariable-adjusted associations of region with components of treatment received for rectal cancer only among AIAN people; Surveillance, Epidemiology and Results program, 2000-2021, n =1,859.

| **Region^a^** | **Treatment Time ≤60 days^b^** | **Microscopic Confirmation** | **Regional Nodes 12 examined** | **MSI testing documented^c^** | **Chemotherapy** | **Radiation** | **Detected at late stage^d^** |
| --- | --- | --- | --- | --- | --- | --- | --- |
| Unadjusted | | | | | | | |
| Alaska | **4.05 (2.78, 5.92)** | 0.64 (0.21, 1.91) | **0.57 (0.44, 0.74)** | **0.55 (0.34, 0.89)** | **0.74 (0.59, 0.93)** | **1.36 (1.08, 1.72)** | 0.86 (0.66, 1.10) |
| East | **2.12 (1.21, 3.69)** | 0.48 (0.10, 2.34) | 1.30 (0.87, 1.94) | 0.59 (0.28, 1.22) | 0.99 (0.67, 1.48) | 0.84 (0.56, 1.25) | 1.37 (0.88, 2.12) |
| Northern Plains | 0.87 (0.40, 1.86) | 133042 (0.00, I) | 1.06 (0.53, 2.14) | 0.66 (0.18, 2.35) | 0.96 (0.48, 1.90) | 1.13 (0.58, 2.23) | 0.88 (0.43, 1.81) |
| Southwest | **2.05 (1.50, 2.80)** | 0.80 (0.25, 2.52) | 0.84 (0.65, 1.07) | **0.43 (0.27, 0.69)** | 0.99 (0.79, 1.25) | 1.10 (0.88, 1.39) | 0.91 (0.71, 1.18) |

Adjusted^e^

| Alaska | **4.28 (2.84, 6.46)** | 0.47 (0.13, 1.74) | **0.59 (0.44, 0.79)** | **0.42 (0.23, 0.76)** | 0.84 (0.63, 1.13) | **1.83 (1.38, 2.43)** | 0.99 (0.75, 1.31) |
| --- | --- | --- | --- | --- | --- | --- | --- |
| East | **1.90 (1.05, 3.45)** | 0.52 (0.08, 3.45) | 1.11 (0.70, 1.77) | 0.72 (0.31, 1.67) | 0.80 (0.48, 1.33) | 0.72 (0.44, 1.17) | 1.41 (0.87, 2.27) |
| Northern Plains | 0.88 (0.40, 1.92) | 206198 (0.00, I) | 1.00 (0.48, 2.09) | 0.77 (0.19, 3.05) | 0.95 (0.42, 2.16) | 1.15 (0.54, 2.46) | 0.83 (0.40, 1.74) |
| Southwest | **2.01 (1.45, 2.79)** | 0.71 (0.20, 2.55) | 0.83 (0.63, 1.09) | **0.33 (0.20, 0.56)** | 1.02 (0.77, 1.35) | 1.19 (0.91, 1.55) | 0.97 (0.74, 1.26) |

^a^Pacific Coast was the region of reference for all analyses. No event was the reference group for all outcomes. Statistically significant associations are given in **bold**.

^b^Patients underwent at least one documented treatment, including chemotherapy, radiation, or cancer-directed surgery. A total of 1,160 individuals were included in these analyses.

^c^MSI testing from 2018 onwards. A total of 467 individuals were included in MSI analyses.

^d^SEER derived summary stage from 2004 onwards. A total of 1,722 individuals were included.

^e^The model was adjusted for covariates including Diagnosis Year, Age Group, Sex, Ethnicity, Marital Status at Diagnosis, stage (except detected at late stage), and PRCDA.

**Supplementary Table 13**. Characteristics of matched AIAN and NHW people with Breast cancer, nationwide, 2000-2021. Data from the Surveillance, Epidemiology, and End Results program, n =77,690.

| **Category** | **AI/AN** **(N=7,108)** | **NHW** **(N=70,582)** |
| --- | --- | --- |
| Diagnosis Age (years, mean (SD)) | | |
|  | 58.5 (13) | 58.4 (13) |
| Age Group (years, %) | | |
| 20-39 | 494 (6.9%) | 4902 (6.9%) |
| 40-64 | 4277 (60.2%) | 42618 (60.4%) |
| 65+ | 2337 (32.9%) | 23062 (32.7%) |
| Income (%) | | |
| < $40,000 | 160 (2.3%) | <5 |
| $40,000-100,000 | 6403 (90.1%) | 36046 (51.1%) |
| >= $100,000 | 541 (7.6%) | 34534 (48.9%) |
| Missing | <5 | 0 (0%) |
| Ethnicity (%) | | |
| Non-Hispanic | 6612 (93%) | 70582 (100%) |
| Hispanic | 496 (7%) | 0 (0%) |
| Marital status at diagnosis (%) | | |
| Not Partnered | 3075 (43.3%) | 26161 (37.1%) |
| Partnered | 3237 (45.5%) | 42386 (60.1%) |
| Unknown | 796 (11.2%) | 2035 (2.9%) |
| RUCC (%) | | |
| Rural | 2575 (36.2%) | 3476 (4.9%) |
| Urban | 4533 (63.8%) | 67106 (95.1%) |
| Diagnosis Year (%) | | |
| 2000-2005 | 1433 (20.2%) | 14308 (20.3%) |
| 2006-2010 | 1455 (20.5%) | 14472 (20.5%) |
| 2011-2015 | 1774 (25%) | 17543 (24.9%) |
| 2016-2021 | 2446 (34.4%) | 24259 (34.4%) |
| PRCDA (%) | | |
| Yes | 5613 (79%) | 28688 (40.6%) |
| No | 1495 (21%) | 41894 (59.4%) |
| CoC Accredited Flag (%) | | |
| Yes | 1070 (63.2%) | 13566 (90.1%) |
| No | 79 (4.7%) | 466 (3.1%) |
| Grade (%) | | |
| Well, differentiated | 1444 (20.3%) | 16931 (24%) |
| Moderately differentiated | 2695 (37.9%) | 29794 (42.2%) |
| Poorly differentiated | 2277 (32%) | 18471 (26.2%) |
| Undifferentiated | 45 (0.6%) | 373 (0.5%) |
| Unknown | 647 (9.1%) | 5013 (7.1%) |
| Stage at Diagnosis (%) | | |
| Local | 3697 (59.7%) | 41014 (68.7%) |
| Regional | 1971 (31.8%) | 15580 (26.1%) |
| Distant | 400 (6.5%) | 2692 (4.5%) |
| Unknown/Unstaged | 121 (2%) | 429 (0.7%) |
| Breast Subtype (%)^a^ |  |  |
| HR+/HER2+ | 505 (11.1%) | 4186 (9.5%) |
| HR+/HER2- | 3044 (67%) | 32442 (73.7%) |
| HR-/HER2+ | 220 (4.8%) | 1615 (3.7%) |
| HR-/HER2- | 466 (10.3%) | 3776 (8.6%) |
| No/Unknown | 309 (6.8%) | 1989 (4.5%) |

^a^Breast cancer subtype/HER2 information available from 2010 onwards. A total of 48,552 individuals were included in these analyses.

**Supplementary Table 14**. Characteristics of matched AIAN and NHW people with lung cancer, nationwide, 200**0**-2021. Data from the Surveillance, Epidemiology, and End Results program, n = 59,392.

| **Category** | **AIAN**  **(N=5,403)** | **NHW**  **(N=53,989)** |
| --- | --- | --- |
| Diagnosis Age (years, mean (SD)) | | |
|  | 68 (10.7) | 68 (10.6) |
| Age Group (%) | | |
| 20-39 | 41 (0.8%) | 369 (0.7%) |
| 40-64 | 1971 (36.5%) | 19710 (36.5%) |
| 65+ | 3391 (62.8%) | 33910 (62.8%) |
| PRCDA Region (%) | | |
| Alaska | 1423 (26.3%) | 0 (0%) |
| East | 449 (8.3%) | 24886 (46.1%) |
| Northern Plains | 107 (2%) | 2703 (5%) |
| Pacific Coast | 2957 (54.7%) | 26330 (48.8%) |
| Southwest | 467 (8.6%) | 70 (0.1%) |
| Sex (%) | | |
| Male | 2742 (50.7%) | 27409 (50.8%) |
| Female | 2661 (49.3%) | 26580 (49.2%) |
| Ethnicity (%) | | |
| Non-Hispanic | 5191 (96.1%) | 53989 (100%) |
| Hispanic | 212 (3.9%) | 0 (0%) |
| Marital status at diagnosis (%) | | |
| Not Partnered | 2684 (49.7%) | 24184 (44.8%) |
| Partnered | 2150 (39.8%) | 27940 (51.8%) |
| Unknown | 569 (10.5%) | 1865 (3.5%) |
| Diagnosis Year (%) | | |
| 2000-2005 | 1193 (22.1%) | 11930 (22.1%) |
| 2006-2010 | 1147 (21.2%) | 11458 (21.2%) |
| 2011-2015 | 1291 (23.9%) | 12899 (23.9%) |
| 2016-2021 | 1772 (32.8%) | 17702 (32.8%) |
| CoC Accredited Flag^a^ (%) | | |
| Yes | 635 (53.2%) | 8774 (83.9%) |
| No | 102 (8.5%) | 924 (8.8%) |
| Grade^b^ (%) | | |
| Well, differentiated | 239 (4.4%) | 3433 (6.4%) |
| Moderately differentiated | 692 (12.8%) | 8196 (15.2%) |
| Poorly differentiated | 1104 (20.4%) | 11662 (21.6%) |
| Undifferentiated | 186 (3.4%) | 1986 (3.7%) |
| Unknown | 3182 (58.9%) | 28712 (53.2%) |
| Stage^c^ (%) | | |
| In situ | 0 (0%) | <5 |
| Localized | 829 (17.8%) | 10543 (23.4%) |
| Regional | 1028 (22%) | 10067 (22.4%) |
| Distant | 2563 (54.9%) | 23432 (52.1%) |
| Unknown/Unstaged | 245 (5.3%) | 941 (2.1%) |

^a^CoC Accredited Flag available from 2018 onwards. A total of 11,648 individuals were included in this analysis.

^b^From 2000 to 2017, variable Grade Recode was used to define grade; from 2018 to present, pathological grade was used.

^c^SEER Combined Stage available from 2004 onwards. A total of 49,650 individuals were included in this analysis.

**Supplementary Table 15.** Characteristics of matched AIAN and NHW people with colorectal cancer, nationwide, 2000-2021. Data from the Surveillance, Epidemiology, and End Results program, n = 61,865.

| **Category** | **AI/AN** **(N=5625)** | **NHW** **(N=56240)** |
| --- | --- | --- |
| Diagnosis Age (y, (mean (SD))) | | |
|  | 5625 62.7 (13.6) | 56240 62.8 (13.6) |
| Age Group (%) | | |
| 20-39 | 295 (5.2%) | 2940 (5.2%) |
| 40-64 | 2730 (48.5%) | 27300 (48.5%) |
| 65+ | 2600 (46.2%) | 26000 (46.2%) |
| PRCDA Region (%) | | |
| Alaska | 1472 (26.2%) | 0 (0%) |
| East | 355 (6.3%) | 19087 (33.9%) |
| Northern Plains | 80 (1.4%) | 9693 (17.2%) |
| Pacific Coast | 2378 (42.3%) | 25524 (45.4%) |
| Southwest | 1340 (23.8%) | 1936 (3.4%) |
| Sex (%) | | |
| Male | 2837 (50.4%) | 28367 (50.4%) |
| Female | 2788 (49.6%) | 27873 (49.6%) |
| Income (%) | | |
| < $40,000 | 167 (3%) | 67 (0.1%) |
| $40,000-100,000 | 5140 (91.4%) | 36645 (65.2%) |
| >= $100,000 | 314 (5.6%) | 19524 (34.7%) |
| Missing | <5 | <5 |
| Ethnicity (%) | | |
| Non-Hispanic | 5246 (93.3%) | 56240 (100%) |
| Hispanic | 379 (6.7%) | 0 (0%) |
| Marital status at diagnosis (%) | | |
| Not Partnered | 2408 (42.8%) | 21814 (38.8%) |
| Partnered | 2375 (42.2%) | 31877 (56.7%) |
| Unknown | 842 (15%) | 2549 (4.5%) |
| RUCC (%) | | |
| Rural | 2652 (47.1%) | 7672 (13.6%) |
| Urban | 2973 (52.9%) | 48568 (86.4%) |
| Diagnosis Year (%) | | |
| 2000-2005 | 1091 (19.4%) | 10910 (19.4%) |
| 2006-2010 | 1143 (20.3%) | 11427 (20.3%) |
| 2011-2015 | 1447 (25.7%) | 14463 (25.7%) |
| 2016-2021 | 1944 (34.6%) | 19440 (34.6%) |
| CoC Accredited Flag (%)^a^ | | |
| Yes | 696 (53.3%) | 9594 (79.8%) |
| No | 57 (4.4%) | 619 (5.1%) |
| Grade (%)^b^ | | |
| Well, differentiated | 661 (11.8%) | 6212 (11%) |
| Moderately differentiated | 3168 (56.3%) | 31927 (56.8%) |
| Poorly differentiated | 658 (11.7%) | 8445 (15%) |
| Undifferentiated | 78 (1.4%) | 846 (1.5%) |
| Unknown | 1060 (18.8%) | 8810 (15.7%) |
| Staged (%)^c^ | | |
| In situ | 0 (0%) | <5 |
| Localized | 1763 (35.7%) | 19304 (40.1%) |
| Regional | 1782 (36.1%) | 17364 (36%) |
| Distant | 1156 (23.4%) | 10168 (21.1%) |
| Unknown/Unstaged | 239 (4.8%) | 1342 (2.8%) |

^a^CoC Accredited Flag available from 2018 onwards. A total of 10,966 individuals were included in this analysis.

^b^From 2000 to 2017, variable Grade Recode was used to define grade; from 2018 to present, pathological grade was used.

^c^SEER Combined Stage available from 2004 onwards. A total of 53,119 individuals were included in this analysis.

**Supplementary Table 16.** Frequencies of individuals receiving components of care among matched AIAN and NHW people with breast cancer (females only), nationwide, 2000-2021, Data from the Surveillance, Epidemiology, and End Results program, n =77,690

| **Category** | **AI/AN**  **(N=7,108)** | **NWH**  **(N=70,582)** |
| --- | --- | --- |
| Time to Treatment [IQR]^a^ | | |
|  | 28 [14, 46] | 27 [14, 40] |
| Time to Treatment ≤60 days (%)^a^ | | |
| Yes | 5311 (79.6%) | 61004 (89.3%) |
| No | 871 (13.1%) | 5590 (8.2%) |
| Unknown | 489 (7.3%) | 1691 (2.5%) |
| Microscopic Confirmation (%) | | |
| Yes | 7066 (99.4%) | 70427 (99.8%) |
| No/Unknown | 42 (0.6%) | 155 (0.2%) |
| Chemotherapy (%)^b^ | | |
| Yes | 3170 (44.6%) | 28374 (40.2%) |
| No/Unknown | 3938 (55.4%) | 42208 (59.8%) |
| Radiation (%)^b^ | | |
| Yes | 3476 (48.9%) | 40963 (58%) |
| No/Unknown | 3632 (51.1%) | 29619 (42%) |
| Cancer-directed Surgery (%) | | |
| Surgery performed | 6405 (90.1%) | 65920 (93.4%) |
| Not recommended | 503 (7.1%) | 3517 (5%) |
| Recommended but not performed | 176 (2.5%) | 1068 (1.5%) |
| Unknown | 24 (0.3%) | 77 (0.1%) |
| Receipt of Surgery (%) | | |
| Surgery received | 6394 (90%) | 65844 (93.3%) |
| No/Unknown | 714 (10%) | 4738 (6.7%) |
| Type of Surgery (%) | | |
| Lumpectomy, or other breast preserving surgery | 2210 (34.6%) | 16557 (25.1%) |
| Mastectomy with or without reconstruction | 3557 (55.6%) | 42302 (64.2%) |
| Bi-lateral Mastectomy with or without reconstruction | 627 (9.8%) | 6985 (10.6%) |
| Breast Cancer Subtype Known (%) |  |  |
| Yes | 4235 (93.2%) | 42019 (95.5%) |
| No | 309 (6.8%) | 1989 (4.5%) |
| ER/PR testing occurred (%) | | |
| Yes | 6662 (93.7%) | 68054 (96.4%) |
| No/Unknown | 446 (6.3%) | 2528 (3.6%) |
| HER2 testing occurred (%)^c^ | | |
| Yes | 4244 (93.4%) | 42087 (95.6%) |
| No/Unknown | 300 (6.6%) | 1921 (4.4%) |
| Diagnosed at late stage (%) | | |
| Yes | 2371 (38.3%) | 18272 (30.6%) |
| No | 3697 (59.7%) | 41014 (68.7%) |
| Unknown | 121 (2%) | 429 (0.7%) |

^a^Patients received at least one documented treatment, including chemotherapy, radiation, or cancer-directed surgery. A total of 74,956 individuals were included in this analysis.

^b^Due to known issues of sensitivity in the chemotherapy and radiation variables (Noone et al. 2016), it is not recommended to interpret these data as reflecting the prevalence of treatment receipt.

**Supplementary Table 17**. Frequencies of individuals receiving components of care among matched AIAN and NHW people with lung cancer, nationwide, 2000-2021. Data from the Surveillance, Epidemiology, and End Results program, n = 59,392.

| **Category** | **AI/AN  (N=5,403)** | **NHW  (N=53,989)** |
| --- | --- | --- |
| Time to Treatment [IQR]^a^ | | |
|  | 29[12, 56] | 27[9, 49] |
| Time to Treatment ≤60 days^a^ | | |
| Yes | 2478 (69.6%) | 32209 (77.9%) |
| No | 702 (19.7%) | 6917 (16.7%) |
| Unknown | 382 (10.7%) | 2234 (5.4%) |
| Microscopic Confirmation (%) | | |
| Yes | 4777 (88.4%) | 50582 (93.7%) |
| No/Unknown | 626 (11.6%) | 3407 (6.3%) |
| Chemotherapy (%)^b^ | | |
| Yes | 2158 (39.9%) | 23630 (43.8%) |
| No/Unknown | 3245 (60.1%) | 30359 (56.2%) |
| Radiation (%)^b^ | | |
| Yes | 2089 (38.7%) | 21127 (39.1%) |
| No/Unknown | 3314 (61.3%) | 32862 (60.9%) |
| Cancer-directed Surgery (%) | | |
| Surgery performed | 928 (17.2%) | 14427 (26.7%) |
| Not recommended | 4030 (74.6%) | 36582 (67.8%) |
| Recommended but not performed | 383 (7.1%) | 2808 (5.2%) |
| Unknown | 62 (1.1%) | 172 (0.3%) |
| Diagnosed at late stage^c^ (%) | | |
| Yes | 3591 (77%) | 33499 (74.5%) |
| No | 829 (17.8%) | 10543 (23.4%) |
| Unknown | 245 (5.3%) | 943 (2.1%) |

^a^Patients underwent at least one documented treatment, including chemotherapy, radiation, cancer-directed surgery. A total of 44,922 individuals were included in this analysis.

^b^Due to known issues of sensitivity in the chemotherapy and radiation variables (Noone et al. 2016), it is not recommended to interpret these data as reflecting prevalence of treatment receipt.

^c^SEER Combined Stage available from 2004. A total of 49,650 participants were included in this analysis.

**Supplementary Table 18**. Frequencies of individuals receiving components of care among matched AIAN and NHW people with colorectal cancer, nationwide, 2000-2021. Data from the Surveillance, Epidemiology, and End Results program, n = 61,865.

| **Category** | **AI/AN (N=5625)** | **NWH (N=56240)** |
| --- | --- | --- |
| Time to Treatment [IQR]^a^ | | |
|  | 13 [0, 33] | 13 [0, 29] |
| Time to Treatment ≤60 days (%)^a^ | | |
| Yes | 4276 (85.4%) | 47185 (90.5%) |
| No | 401 (8%) | 2679 (5.1%) |
| Unknown | 328 (6.6%) | 2285 (4.4%) |
| Microscopically Confirmation (%) | | |
| Yes | 5484 (97.5%) | 55551 (98.8%) |
| No/Unknown | 141 (2.5%) | 689 (1.2%) |
| Chemotherapy (%)^b^ | | |
| Yes | 2321 (41.3%) | 23565 (41.9%) |
| No/Unknown | 3304 (58.7%) | 32675 (58.1%) |
| Radiation (%)^b^ | | |
| Yes | 974 (17.3%) | 8118 (14.4%) |
| No/Unknown | 4651 (82.7%) | 48122 (85.6%) |
| Cancer-directed Surgery (%) | | |
| Surgery performed | 4453 (79.2%) | 47052 (83.7%) |
| Not recommended | 1172 (20.8%) | 9188 (16.3%) |
| Receipt of Surgery-Colon (%) | | |
| Yes | 3022 (82.6%) | 31553 (86.9%) |
| No/Unknown | 635 (17.4%) | 4755 (13.1%) |
| Type of Surgery-Colon (%)^c^ | | |
| Minor surgery | 152 (4.2%) | 1940 (5.3%) |
| Partial colectomy | 1177 (32.2%) | 11942 (32.9%) |
| Subtotal or total colectomy | 1693 (46.3%) | 17671 (48.7%) |
| No/Unknown | 635 (17.4%) | 4755 (13.1%) |
| Receipt of Surgery-Rectum (%) | | |
| Yes | 1328 (71.5%) | 13497 (76.1%) |
| No/Unknown | 530 (28.5%) | 4238 (23.9%) |
| Type of Surgery-Rectum (%)^c^ | | |
| Minor surgery | 312 (16.8%) | 3044 (17.2%) |
| Wedge or segmental resection; pull through with sphincter preservation | 799 (43%) | 8361 (47.1%) |
| Total proctectomy or proctolectomy | 217 (11.7%) | 2092 (11.8%) |
| No/Unknown | 530 (28.5%) | 4238 (23.9%) |
| Regional Nodes 12+ examined (%) | | |
| Yes | 2721 (48.4%) | 31378 (55.8%) |
| No/Unknown | 2904 (51.6%) | 24862 (44.2%) |
| KRAS testing (%)^d^ | | |
| Abnormal | 59 (19.5%) | 540 (20.9%) |
| Missing or Invalid | 7 (2.3%) | 182 (7%) |
| Normal (wild type) | 73 (24.2%) | 572 (22.2%) |
| Unknown | 163 (54%) | 1288 (49.9%) |
| KRAS testing documented (%)^d^ | | |
| Yes | 132 (43.7%) | 1112 (43.1%) |
| No/Unknown | 170 (56.3%) | 1470 (56.9%) |
| MSI testing(%)^d^ | | |
| MSI Stable | 611 (46.8%) | 6973 (58%) |
| MSI Unstable | 90 (6.9%) | 1170 (9.7%) |
| Missing or Invalid | 78 (6%) | 1026 (8.5%) |
| Unknown | 526 (40.3%) | 2855 (23.7%) |
| MSI testing documented (%)^d^ | | |
| Yes | 701 (53.7%) | 8143 (67.7%) |
| No/Unknown | 604 (46.3%) | 3881 (32.3%) |
| Diagnosed at late stage (%)^e^ | | |
| Yes | 2938 (59.5%) | 27532 (57.1%) |
| No | 1763 (35.7%) | 19304 (40.1%) |
| Unknown | 239 (4.8%) | 1343 (2.8%) |

^a^Patients underwent at least one documented treatment, including chemotherapy, radiation, cancer-directed surgery. A total of 57154 individuals were included.

^b^Due to known issues of sensitivity in the chemotherapy and radiation variables (Noone et al. 2016), it is not recommended to interpret these data as reflecting prevalence of treatment receipt.

^c^A total of 39965 patients with colon cancer were included, and a total of 19,593 with rectal cancer.

^d^KRAS and MSI status available from 2018 onwards. Results of KRAS testing includes stage IV only. A total of 2,884 individuals were included in KRAS analysis, and 13,329 participants were included in the MSI analysis.

^e^SEER Combined Stage available from 2004. A total of 53,119 participants were included in this analysis.

**Supplementary Table 19**. Multivariable-adjusted associations of race with components of treatment received for breast cancer among matched AIAN and NHW people, females only, nationwide, 2010-2021. Data from the Surveillance, Epidemiology, and End Results program, n =48,552.^a^

|  | Unadjusted | | Adjusted^b^ | |
| --- | --- | --- | --- | --- |
| Category | OR (95% CI) | P value | aOR (95% CI) | P value |
| Time to Treatment ≤60 days^c^ | **0.53 (0.49, 0.58)** | <.0001 | **0.53 (0.46, 0.62)** | <.0001 |
| Receipt of Chemotherapy | **1.23 (1.15, 1.31)** | <.0001 | 1.08 (0.94, 1.25) | 0.2753 |
| Receipt of Radiation | **0.69 (0.65, 0.74)** | <.0001 | **0.73 (0.65, 0.81)** | <.0001 |
| Lumpectomy, or other breast-preserving surgery^d^ | **0.74 (0.69, 0.79)** | <.0001 | **0.81 (0.72, 0.91)** | 0.0006 |
| HER2 testing occurred | **0.65 (0.57, 0.73)** | <.0001 | 0.81 (0.62, 1.06) | 0.1248 |
| ER/PR testing occurred | **0.36 (0.30, 0.42)** | <.0001 | 0.79 (0.51, 1.22) | 0.2858 |
| Diagnosed at late stage^e^ | **1.46 (1.37, 1.55)** | <.0001 | **1.44 (1.30, 1.61)** | <.0001 |

^a^Reference groups were no event for outcome variables and non-Hispanic white for the primary independent variable (i.e., race).

^b^Models were adjusted for covariates including PRCDA region, PRCDA residence, marital status, breast subtype (except HER2 and ER/PR models) and cancer stage at diagnosis (except diagnosed at late stage model).

^c^Patients underwent at least one documented treatment, including chemotherapy, radiation, or cancer-directed surgery. A total of 46,569 individuals were included in this analysis.

^d^All patients included in this analysis received surgery. A total of 44,632 individuals were included in this analysis.

^e^Late stage diagnosis was defined as diagnosis at regional or distant stage.

**Supplementary Table 20.** Multivariable-adjusted associations of race with components of treatment received for lung cancer among matched AIAN and NHW, nationwide, [2000-2021]. Data from the Surveillance, Epidemiology, and End Results program, n = 59,392.^a^

|  | **Unadjusted** | | **Adjusted^b^** | |
| --- | --- | --- | --- | --- |
| **Category** | **OR (95%CI)** | **P value** | **aOR (95%CI)** | **P value** |
| Time to Treatment ≤60 days^c^ | **0.65 (0.60, 0.70)** | <.0001 | **0.73 (0.64, 0.84)** | <.0001 |
| Microscopic Confirmation | **0.51 (0.47, 0.56)** | <.0001 | **0.63 (0.53, 0.74)** | <.0001 |
| Receipt of Chemotherapy | **0.85 (0.81, 0.90)** | <.0001 | **0.83 (0.75, 0.91)** | 0.0002 |
| Receipt of Radiation | 0.98 (0.93, 1.04) | 0.5019 | **0.88 (0.80, 0.97)** | 0.0097 |
| Receipt of Surgery | **0.57 (0.53, 0.61)** | <.0001 | **0.70 (0.61, 0.80)** | <.0001 |
| Detected at late stage^d^ | **1.36 (1.26, 1.47)** | <.0001 | **1.19 (1.05, 1.34)** | 0.0058 |

^a^Reference groups were no event for outcome variables and Non-Hispanic White for the primary independent variable (i.e., race).

^b^The model was adjusted for covariates including PRCDA region, PRCDA residence, marital status, and cancer stage at diagnosis (except detected at late stage).

^c^Patients underwent at least one documented treatment, including chemotherapy, radiation, or cancer-directed surgery. A total of 44,922 individuals were included.

^d^SEER Combined Stage available from 2004 onwards. A total of 49,650 individuals with stage information were included in this analysis. Late stage diagnosis was defined as diagnosis at regional or distant stage.

**Supplementary Table 21**. Multivariable-adjusted associations of race with components of treatment received for colorectal cancer among matched AIAN and NHW, nationwide, 2000-2021. Data from the Surveillance, Epidemiology, and End Results program, n = 61,865.^a^

|  | **Unadjusted** | | **Adjusted^b^** | |
| --- | --- | --- | --- | --- |
| **Category** | **OR (95% CI)** | **P value** | **aOR (95% CI)** | **P value** |
| Time to Treatment ≤60 days (%)^c^ | 0.60 (0.56, 0.64) | <.0001 | **0.50 (0.44, 0.57)** | <.0001 |
| Microscopic Confirmation | 0.48 (0.40, 0.58) | <.0001 | **0.60 (0.41, 0.87)** | 0.0073 |
| Receipt of Chemotherapy | 0.97 (0.92, 1.03) | 0.3555 | 0.97 (0.88, 1.08) | 0.6126 |
| Receipt of Radiation | 1.24 (1.15, 1.34) | <.0001 | **1.25 (1.12, 1.39)** | <.0001 |
| Receipt of Surgery Colon^d^ | 0.72 (0.65, 0.79) | <.0001 | **0.80 (0.65, 0.97)** | 0.0211 |
| Receipt of Surgery Rectum^d^ | 0.79 (0.71, 0.88) | <.0001 | 0.92 (0.74, 1.14) | 0.4355 |
| 12 Regional Lymph Nodes Examined | 0.74 (0.70, 0.78) | <.0001 | **0.83 (0.77, 0.91)** | <.0001 |
| MSI testing documented^e^ | 0.55 (0.49, 0.62) | <.0001 | **0.72 (0.60, 0.87)** | 0.0005 |
| Diagnosed at late stage^f^ | 1.17 (1.10, 1.24) | <.0001 | **1.21 (1.10, 1.32)** | <.0001 |

^a^Reference groups were no event for outcome variables and non-Hispanic white for the primary independent variable (i.e., race). Analyses of KRAS not shown in these tables due to small numbers, and high proportions (>95%) missing data.

^b^Models were adjusted for covariates including PRCDA region, PRCDA residence, marital status, and cancer stage at diagnosis.

^c^Patients received at least one documented treatment, including chemotherapy, radiation, or cancer-directed surgery. A total of 57,154 individuals were included in this analysis.

^d^A total of 39,965 patients with colon cancer were included, and a total of 19,593 with rectal cancer were included in these analyses.

^e^MSI status available from 2018 onwards. A total of 13,329 participants were included in the MSI analysis.

^f^SEER Combined Stage available from 2004 onwards. A total of 51,537 individuals with stage results were included in this analysis.
